# Supplementary material for: Validity conditions of approximations for a target-mediated drug disposition model: A novel first-order approximation and its comparison to other approximations
Source: PLoS Comput Biol. 2024 Apr 24;20(4):e1012066. doi: 10.1371/journal.pcbi.1012066 (PMC11090311; doi:10.1371/journal.pcbi.1012066)
Supplement: S1 Text — (DOCX) [file pcbi.1012066.s001.docx]

**S1 Text. The NONMEM code of the population pharmacokinetic model with each approximation method implemented.**

1. Dataset for case 1 (*hil-1-ra-hyfc*) was attached as an electrical file named by S1 Table. Dataset for case 1 (hil-1ra-hyfc).csv, and the original TMDD model for case 1 (*hil-1-ra-hyfc*) is as follows:

$PROBLEM 1st-order 2cmt

;; 1. Based on: original

;; 2. Description: original

;; x1. Author: hyeseon

$DATA S1 Table. Dataset for case 1 (hil-1ra-hyfc).csv IGNORE=@

$INPUT ID TIME AMT CMT DV LNDV MDV EVID

$SUBROUTINE ADVAN13 TOL=6

$MODEL NCOMP=6 COMP=(DEPOT,DEFDOSE) COMP=(ABS) COMP=(CENTRAL)

COMP=(PERIPH) COMP=(CPX1) COMP=(CPX2)

$PK

Ka1 = THETA(1)* EXP(ETA(1)) ; Absorption rate constant from injection site

KON1 = THETA(2)

FcRn = THETA(3) ; Total amount (unbound- and bound-to HL2351) of FcRn

Ka2 = THETA(4)* EXP(ETA(2)) ; Transportation rate constant of free HL2351 from distribution space to central compartment

Krec = THETA(5)* EXP(ETA(3)) ; Recycling rate constant of HL2351 bound to FcRn from distribution space to central compartment

Kdeg1= THETA(6)* EXP(ETA(4)) ; Degradation rate constant of free HL2351 in distribution space

CL = THETA(7)* EXP(ETA(5)) ; Apparent clearance of free HL2351 from central compartment

V3 = THETA(8) ; Apparent volume of distribution of free HL2351 in central compartment

Q = THETA(9) ; Apparent inter-compartment clearance of free HL2351 between central and peripheal compartments

V4 = THETA(10) ; Apparent volume of distribution of free HL2351 in peripheral compartment

Kdeg2= THETA(11) ; Degradation rate constant of free HL2351 in central compartment

Rtot = THETA(12) ; Total amount (unbound- and bound-to HL2351) of IL1R

KON2 = THETA(13)

Kup = THETA(14)* EXP(ETA(6)) ; Endosomal uptake rate constant of free HL2351 from central compartment to distribution space

Alag1= THETA(15)* EXP(ETA(7)) ; Lag time for drug absorption

S2 = V3

S3 = V4

Kel = CL/V3

Kpt = Q/V3

Ktp = Q/V4

KOFF1 = THETA(18)

KOFF2 = THETA(19)

;V3_CPX=THETA(20)

V2 =THETA(20)

$DES

Afree = A(2)/V2

Cfree = A(3)/V3

; Injection site

DADT(1) = -Ka1*A(1)

; Distribution space-free HL2351 amount (nmol)

DADT(2) = Ka1*A(1) - (Kdeg1 + Ka2)*A(2) - KON1*Afree*FcRn + (KON1*Afree + KOFF1)*A(5) + Kup*A(3)

; Central compartment-Total HL2351 amount (nmol)

DADT(3) = Krec*A(5) + Ka2*A(2) + Ktp*A(4)-(Kel+Kpt)*A(3) - KON2*A(3)*Rtot + (KON2*Cfree + KOFF2) * A(6) - Kup*A(3)

; Peripheral comparment-Free HL2351 amount (nmol)

DADT(4) = Kpt*A(3) - Ktp*A(4)

; Distribution space- CPX HL2351 amount (nmol)

DADT(5) = KON1*Afree*FcRn - (KON1*Afree + KOFF1 + Krec) * A(5)

; Central compartment-CPX HL2351 amount (nmol)

DADT(6) = KON2*A(3)*Rtot - (KON2*Cfree + KOFF2 + Kdeg2) * A(6)

$ERROR

Cf = A(3)/V3

IPRED = Cf

W = SQRT(THETA(16)**2 + THETA(17)**2*IPRED**2)

IRES = DV-IPRED

IWRES = IPRED/W

Y = IPRED + W*EPS(1)

$THETA

(0,1.22) ; Ka1

(0,0.249) ; 2.KON1 (; (pM^-1 h*-1))

(0,749 FIX) ; FcRn nmol

(0,0.0166) ; Ka2 1/h

(0,0.0345) ; Krec 1/h

(0,0.0264 FIX) ; Kdeg1 1/h

(0,0.208) ; CL L/h

(0,11.4) ; V3 L

(0,0.0285) ; Q L/h

5.06 FIX ; V4 L

0.206 FIX ; 11.Kdeg2 1/h

(0,2.23 FIX) ; Rtot nmol/L

(0,6.47) ; 13.KON2 (; (pM^-1 h*-1))

0.00952 FIX ; 14.Kup 1/h

(0,0.241) ; Alag1 h

(0,0.187) ; additive ERROR

(0,0.115) ; proportional ERROR

(0.001,0.466) ; 18.KOFF1 1/h

(0.001,95.4) ; 19.KOFF2 1/h

;(0.001,11.9) ; 20.V3_CPX

(0,117) ; 20.V2

$OMEGA

0.883 ; Ka1_

0.549 ; Ka2

0.0972 ; Krec

0.0867 ; Kdeg1

0.051 ; CL

0.874 ; Kup

0.403 ; Alag1

$SIGMA 1 FIX

$COVARIANCE MATRIX=S PRINT=E

$ESTIMATION NSIG=2 SIGL=6 PRINT=1 METHOD=1 MAX=9999 NOABORT INTER

$TABLE ID TIME IPRED PRED IWRES CWRES ONEHEADER NOPRINT

FILE=sdtab016

$TABLE ID CL V3 Q V4 Kel Kpt Ktp ONEHEADER NOPRINT FILE=patab016

$TABLE ID ETA(1) ETA(2) ETA(3) ETA(4) ETA(5) ONEHEADER FIRSTONLY

NOPRINT NOAPPEND FILE=etatable.txt

;-----------------------

;TIME = h; DOSE = nmol; Cp = nmol/L;

;-----------------------

(b) Dataset for case 1 (*hil-1-ra-hyfc*) was attached as an electrical file named by S1 Table. Dataset for case 1 (hil-1ra-hyfc).csv, and the mTMDD model for case 1 (*hil-1-ra-hyfc*) is as follows:

$PROBLEM    1st-order 2cmt

;; 1. Based on: mtmdd, ver2

;; 2. Description: mtmdd, ver2

;; x1. Author: hyeseon

$DATA      S1 Table. Dataset for case 1 (hil-1ra-hyfc).csv IGNORE=@

$INPUT      ID TIME AMT CMT DV LNDV MDV EVID

            DVMG=DROP

$SUBROUTINE ADVAN13 TOL=6

$MODEL      NCOMP=4 COMP=(DEPOT,DEFDOSE) COMP=(ABS) COMP=(CENTRAL)

            COMP=(PERIPH)

$PK

      Ka1  = THETA(1)* EXP(ETA(1))  ; Absorption rate constant from injection site

      KSS1 = THETA(2)               ; QSS constant for interactions of FcRn and HL2351

      FcRn = THETA(3)               ; Total amount (unbound- and bound-to HL2351) of FcRn

      Ka2  = THETA(4)* EXP(ETA(2))  ; Transportation rate constant of free HL2351 from distribution space to central compartment

      Krec = THETA(5)* EXP(ETA(3))  ; Recycling rate constant of HL2351 bound to FcRn from distribution space to central compartment

      Kdeg1= THETA(6)* EXP(ETA(4))  ; Degradation rate constant of free HL2351 in distribution space

      CL   = THETA(7)* EXP(ETA(5))  ; Apparent clearance of free HL2351 from central compartment

      V3   = THETA(8)               ; Apparent volume of distribution of free HL2351 in central compartment

      Q    = THETA(9)               ; Apparent inter-compartment clearance of free HL2351 between central and peripheal compartments

      V4   = THETA(10)              ; Apparent volume of distribution of free HL2351 in peripheral compartment

      Kdeg2= THETA(11)              ; Degradation rate constant of free HL2351 in central compartment

      Rtot = THETA(12)              ; Total amount (unbound- and bound-to HL2351) of IL1R

      KSS2 = THETA(13)              ; QSS constant for interactions of IL1R and HL2351

      Kup  = THETA(14)* EXP(ETA(6)) ; Endosomal uptake rate constant of free HL2351 from central compartment to distribution space

      Alag1= THETA(15)* EXP(ETA(7)) ; Lag time for drug absorption

      S2   = V3

      S3   = V4

      Kel  = CL/V3

      Kpt  =  Q/V3

      Ktp  =  Q/V4

$DES

    ; QSS approximations for FcRn-HL2351 interaction

        Acpx = FcRn*A(2)/(KSS1+A(2))              ; (nmol)

    ; QSS approximations for IL1R-HL2351 interaction

           Cf =  A(3)/V3                            ; Total concentration of HL2351 in central compartment (nmol/L)

           Ccpx = Rtot*Cf/(KSS2+Cf)                 ; (nmol/L)

    ; Injection site

      DADT(1) = -Ka1*A(1)

    ; Distribution space-Total HL2351 amount   (nmol)

      DADT(2) =  Ka1*A(1) - (Kdeg1 + Ka2)*A(2) - Krec*Acpx + Kup*A(3)

    ; Central compartment-Total HL2351 amount  (nmol)

      DADT(3) =  Krec*Acpx + Ka2*A(2) + Ktp*A(4) - (Kel+Kpt)*A(3) - Kdeg2*Ccpx*V3 - Kup*A(3)

    ; Peripheral comparment-Free HL2351 amount (nmol)

      DADT(4) =  Kpt*A(3) - Ktp*A(4)

$ERROR

        C =  A(3)/V3                            ; Total concentration of HL2351 in central compartment (nmol/L)

        IPRED = C

        W     = SQRT(THETA(16)**2+THETA(17)**2*IPRED**2)

        IRES  = DV-IPRED

        IWRES = IPRED/W

        Y     = IPRED + W*EPS(1)

$THETA

 (0,1.17) ; Ka1

 (0,5.99) ; KSS1  nmol

 (0,749 FIX) ; FcRn  nmol

 (0,0.018) ; Ka2   1/h

 (0,0.0104) ; Krec  1/h

 (0,0.0264 FIX) ; Kdeg1 1/h

 (0,0.21) ; CL    L/h

 (0,11.7) ; V3    L

 (0,0.0145) ; Q     L/h

 5.06 FIX ; V4    L

 0.206 FIX ; Kdeg2 1/h

 (0,2.23 FIX) ; Rtot  nmol/L

 (0,67.8) ; KSS2  nmol/L

 0.00952 FIX ; Kup   1/h

 (0,0.407) ; Alag1 h

 (0,0.237) ; additive ERROR

 (0,0.0987) ; proportional ERROR

$OMEGA

 1.21  ;       Ka1_

 0.36  ;        Ka2

 0.398  ;       Krec

 0.0789  ;      Kdeg1

 0.039  ;         CL

 0.927  ;        Kup

 0.323  ;      Alag1

$SIGMA  1  FIX

$COVARIANCE MATRIX=S PRINT=E

$ESTIMATION NSIG=2 SIGL=6 PRINT=1 METHOD=1 MAX=9999 NOABORT INTER

$TABLE ID TIME IPRED PRED IWRES CWRES ONEHEADER NOPRINT

FILE=sdtab017

$TABLE ID CL V3 Q V4 Kel Kpt Ktp KSS1 KSS2 Cf Ccpx ONEHEADER NOPRINT

FILE=patab017

$TABLE ID ETA(1) ETA(2) ETA(3) ETA(4) ETA(5) ONEHEADER FIRSTONLY

NOPRINT NOAPPEND FILE=etatable.txt

;-----------------------

;TIME = h; DOSE = nmol; Cp = nmol/L;

;-----------------------

(c) Dataset for case 1 (*hil-1-ra-hyfc*) was attached as an electrical file named by S1 Table. Dataset for case 1 (hil-1ra-hyfc).csv, and the qTMDD model for case 1 (*hil-1-ra-hyfc*) is as follows:

$PROBLEM 1st-order 2cmt

;; 1. Based on: qtmdd, ver2

;; 2. Description: qtmdd, ver2

;; x1. Author: hyeseon

$DATA S1 Table. Dataset for case 1 (hil-1ra-hyfc).csv IGNORE=@

$INPUT ID TIME AMT CMT DV LNDV MDV EVID

$SUBROUTINE ADVAN13 TOL=6

$MODEL NCOMP=4 COMP=(DEPOT,DEFDOSE) COMP=(ABS) COMP=(CENTRAL)

COMP=(PERIPH)

$PK

Ka1 = THETA(1)* EXP(ETA(1)) ; Absorption rate constant from injection site

KSS1 = THETA(2) ; QSS constant for interactions of FcRn and HL2351

FcRn = THETA(3) ; Total amount (unbound- and bound-to HL2351) of FcRn

Ka2 = THETA(4)* EXP(ETA(2)) ; Transportation rate constant of free HL2351 from distribution space to central compartment

Krec = THETA(5)* EXP(ETA(3)) ; Recycling rate constant of HL2351 bound to FcRn from distribution space to central compartment

Kdeg1= THETA(6)* EXP(ETA(4)) ; Degradation rate constant of free HL2351 in distribution space

CL = THETA(7)* EXP(ETA(5)) ; Apparent clearance of free HL2351 from central compartment

V3 = THETA(8) ; Apparent volume of distribution of free HL2351 in central compartment

Q = THETA(9) ; Apparent inter-compartment clearance of free HL2351 between central and peripheal compartments

V4 = THETA(10) ; Apparent volume of distribution of free HL2351 in peripheral compartment

Kdeg2= THETA(11) ; Degradation rate constant of free HL2351 in central compartment

Rtot = THETA(12) ; Total amount (unbound- and bound-to HL2351) of IL1R

KSS2 = THETA(13) ; QSS constant for interactions of IL1R and HL2351

Kup = THETA(14)* EXP(ETA(6)) ; Endosomal uptake rate constant of free HL2351 from central compartment to distribution space

Alag1= THETA(15)* EXP(ETA(7)) ; Lag time for drug absorption

S2 = V3

S3 = V4

Kel = CL/V3

Kpt = Q/V3

Ktp = Q/V4

$DES

; QSS approximations for FcRn-HL2351 interaction

DAA = A(2)-FcRn-KSS1 ; (nmol)

Afree = 0.5*(DAA+SQRT(DAA**2+4*KSS1*A(2))) ; Amount of free HL2351 derived from total HL2351 amount in distribution space (nmol)

Acpx = FcRn*Afree/(KSS1+Afree) ; (nmol)

; QSS approximations for IL1R-HL2351 interaction

Ct = A(3)/V3 ; Total concentration of HL2351 in central compartment (nmol/L)

D = Ct-Rtot-KSS2 ; (nmol/L)

CP = 0.5*(D+SQRT(D**2+4*KSS2*Ct)) ; Concentration of free HL2351 derived from total HL2351 concentration in

; central compartment (nmol/L)

Ccpx = Rtot*CP/(KSS2+CP) ; (nmol/L)

; Injection site

DADT(1) = -Ka1*A(1)

; Distribution space-Total HL2351 amount (nmol)

DADT(2) = Ka1*A(1) - (Kdeg1 + Ka2)*Afree - Krec*Acpx + Kup*CP*V3

; Central compartment-Total HL2351 amount (nmol)

DADT(3) = Krec*Acpx + Ka2*Afree + Ktp*A(4) - (Kel+Kpt)*CP*V3 -Kdeg2*Ccpx*V3 - Kup*CP*V3

; Peripheral comparment-Free HL2351 amount (nmol)

DADT(4) = Kpt*CP*V3 - Ktp*A(4)

$ERROR

Ctot = A(3)/V3

DD = Ctot-Rtot-KSS2

Cfree = 0.5*(DD+SQRT(DD**2+4*KSS2*Ctot))

IPRED = Cfree

W = SQRT(THETA(16)**2+THETA(17)**2*IPRED**2)

IRES = DV-IPRED

IWRES = IPRED/W

Y = IPRED + W*EPS(1)

$THETA (0,1.19) ; Ka1

(0,227) ; KSS1 nmol

(0,749 FIX) ; FcRn nmol

(0,0.017) ; Ka2 1/h

(0,0.0331) ; Krec 1/h

(0,0.0264 FIX) ; Kdeg1 1/h

(0,0.208) ; CL L/h

(0,11.1) ; V3 L

(0,0.0292) ; Q L/h

5.06 FIX ; V4 L

0.206 FIX ; Kdeg2 1/h

(0,2.23 FIX) ; Rtot nmol/L

(0,14) ; KSS2 nmol/L

0.00952 FIX ; Kup 1/h

(0,0.304) ; Alag1 h

(0,0.184) ; additive ERROR

(0,0.115) ; proportional ERROR

$OMEGA

0.927 ; Ka1_

0.524 ; Ka2

0.109 ; Krec

0.0752 ; Kdeg1

0.0535 ; CL

0.801 ; Kup

0.319 ; Alag1

$SIGMA 1 FIX

$COVARIANCE MATRIX=S PRINT=E

$ESTIMATION NSIG=2 SIGL=6 PRINT=1 METHOD=1 MAX=9999 NOABORT INTER

$TABLE ID TIME IPRED PRED IWRES CWRES ONEHEADER NOPRINT

FILE=sdtab011

$TABLE ID CL V3 Q V4 Kel Kpt Ktp KSS1 KSS2 ONEHEADER NOPRINT

FILE=patab011

$TABLE ID ETA(1) ETA(2) ETA(3) ETA(4) ETA(5) ONEHEADER FIRSTONLY

NOPRINT NOAPPEND FILE=etatable.txt

;-----------------------

;TIME = h; DOSE = nmol; Cp = nmol/L;

;-----------------------

(d) Dataset for case 1 (*hil-1-ra-hyfc*) was attached as an electrical file named by S1 Table. Dataset for case 1 (hil-1ra-hyfc).csv, and the pTMDD model for case 1 (*hil-1-ra-hyfc*) is as follows:

$PROBLEM    1st-order 2cmt

;; 1. Based on: tTMDD, ver2

;; 2. Description: tTMDD, ver2

;; x1. Author: hyeseon

$DATA      S1 Table. Dataset for case 1 (hil-1ra-hyfc).csv IGNORE=@

$INPUT      ID TIME AMT CMT DV LNDV MDV EVID $SUBROUTINE ADVAN13 TOL=6

$MODEL      NCOMP=4 COMP=(DEPOT,DEFDOSE) COMP=(ABS) COMP=(CENTRAL)

            COMP=(PERIPH)

$PK

      Ka1  = THETA(1)* EXP(ETA(1))  ; Absorption rate constant from injection site

      KSS1 = THETA(2)               ; QSS constant for interactions of FcRn and HL2351

      FcRn = THETA(3)               ; Total amount (unbound- and bound-to HL2351) of FcRn

      Ka2  = THETA(4)* EXP(ETA(2))  ; Transportation rate constant of free HL2351 from distribution space to central compartment

      Krec = THETA(5)* EXP(ETA(3))  ; Recycling rate constant of HL2351 bound to FcRn from distribution space to central compartment

      Kdeg1= THETA(6)* EXP(ETA(4))  ; Degradation rate constant of free HL2351 in distribution space

      CL   = THETA(7)* EXP(ETA(5))  ; Apparent clearance of free HL2351 from central compartment

      V3   = THETA(8)               ; Apparent volume of distribution of free HL2351 in central compartment

      Q    = THETA(9)               ; Apparent inter-compartment clearance of free HL2351 between central and peripheral compartments

      V4   = THETA(10)              ; Apparent volume of distribution of free HL2351 in peripheal compartment

      Kdeg2= THETA(11)              ; Degradation rate constant of free HL2351 in central compartment

      Rtot = THETA(12)              ; Total amount (unbound- and bound-to HL2351) of IL1R

      KSS2 = THETA(13)              ; QSS constant for interactions of IL1R and HL2351

      Kup  = THETA(14)* EXP(ETA(6)) ; Endosomal uptake rate constant of free HL2351 from central compartment to distribution space

      Alag1= THETA(15)* EXP(ETA(7)) ; Lag time for drug absorption

      S2   = V3

      S3   = V4

      Kel  = CL/V3

      Kpt  =  Q/V3

      Ktp  =  Q/V4

$DES

    ; QSS approximations for FcRn-HL2351 interaction

        Acpx = FcRn*A(2)/(KSS1+A(2)+FcRn)              ; (nmol)

        Afree = A(2) - Acpx

    ; QSS approximations for IL1R-HL2351 interaction

           Ct =  A(3)/V3                            ; Total concentration of HL2351 in central compartment (nmol/L)

           Ccpx = Rtot*Ct/(KSS2+Ct+Rtot)                 ; (nmol/L)

           Cfree = Ct - Ccpx

    ; Injection site

      DADT(1) = -Ka1*A(1)

    ; Distribution space-Total HL2351 amount   (nmol)

      DADT(2) =  Ka1*A(1) - (Kdeg1 + Ka2)*Afree - Krec*Acpx + Cfree*Kup*V3

    ; Central compartment-Total HL2351 amount  (nmol)

      DADT(3) =  Krec*Acpx + Ka2*Afree + Ktp*A(4)-(Kel+Kpt)*Cfree*V3 -Kdeg2*Ccpx*V3 - Cfree*Kup*V3

    ; Peripheral comparment-Free HL2351 amount (nmol)

      DADT(4) =  Kpt*Cfree*V3 - Ktp*A(4)

$ERROR

        Ctot =  A(3)/V3                            ; Total concentration of HL2351 in central compartment (nmol/L)

        Ccp = Rtot*Ctot/(KSS2+Ctot+Rtot)                 ; (nmol/L)

        Cf = Ctot - Ccp

        IPRED = Cf

        W     = SQRT(THETA(16)**2+THETA(17)**2*IPRED**2)

        IRES  = DV-IPRED

        IWRES = IPRED/W

        Y     = IPRED + W*EPS(1)

$THETA

 (0,1.24) ; Ka1

 (0,162) ; KSS1  nmol

 (0,749) FIX ; FcRn  nmol

 (0,0.0156) ; Ka2   1/h

 (0,0.0403) ; Krec  1/h

 (0,0.0264) FIX ; Kdeg1 1/h

 (0,0.199) ; CL    L/h

 (0,11.3) ; V3    L

 (0,0.0279) ; Q     L/h

 5.06 FIX ; V4    L

 0.206 FIX ; Kdeg2 1/h

 (0,2.23) FIX ; Rtot  nmol/L

 (0,14.7) ; KSS2  nmol/L

 0.00952 FIX ; Kup   1/h

 (0,0.326) ; Alag1 h

 (0,0.185) ; additive ERROR

 (0,0.116) ; proportional ERROR

$OMEGA

 1.000  ;       Ka1_

 0.549  ;        Ka2

 0.0978  ;       Krec

 0.0638  ;      Kdeg1

 0.0554  ;         CL

 0.829  ;        Kup

 0.302  ;      Alag1

$SIGMA  1  FIX

$COVARIANCE MATRIX=S PRINT=E

$ESTIMATION NSIG=2 SIGL=6 PRINT=1 METHOD=1 MAX=9999 NOABORT INTER

$TABLE ID TIME IPRED PRED IWRES CWRES ONEHEADER NOPRINT

FILE=sdtab015

$TABLE ID CL V3 Q V4 Kel Kpt Ktp KSS1 KSS2 ONEHEADER NOPRINT

FILE=patab015

$TABLE ID ETA(1) ETA(2) ETA(3) ETA(4) ETA(5) ONEHEADER FIRSTONLY

NOPRINT NOAPPEND FILE=etatable.txt

;-----------------------

;TIME = h; DOSE = nmol; Cp = nmol/L;

;-----------------------

(e) Dataset for case 2 (*rhil-7-hyfc*) was attached as an electrical file named by case2_rhil-7-hyfc.csv, and the original TMDD model for case 2 (*rhil-7- hyfc*) is as follows:

$PROBLEM    GX-I7 FCRN TMDD PK

;; 1. Based on: run009_0314

;; 2. Description: original

;; x1. Author: hyeseon

$INPUT      ID TIME AMT DV MDV COHORT CMT BASE

$DATA      case2_rhil-7-hyfc.csv IGNORE=#

$SUBROUTINE ADVAN13 TOL=6

$MODEL      COMP(IMDEPOT) COMP(SCDEPOT) COMP(CENTRAL)

            COMP(DISTRIBUTION1) ; Absorption site Interstitial & Vascular

            COMP(DISTRIBUTION2) ; FcRn recycling space

            COMP(CPX1) COMP(CPX2)

$PK

F1 = 2

F2 = 2

;--- Central compartment [3]

CL = THETA(1) ; Cl central

VC = THETA(2) ; Vd central

KEL = CL/VC

;--- Injection depot [1] [2]

KAIM = THETA(3) ; K Absorption IM

KASC = THETA(4) ; K Absorption SC

;--- Interstitial& Vascular [4]

Kup = 0.00952              ; uptake rate to endosomal space (ref)

IF(TIME.EQ.0) Kup=0

Q = THETA(5) * EXP(ETA(1)) ; Intercompartmental clearance

;for IM

VIIM = THETA(13)           ; Volume of distribution 1 of interstitial&vascular

;for SC

VISC = THETA(14)

VI=VISC

IF(COHORT.EQ.3) VI=VIIM

K43 = Q/VI

K34 = Q/VC

;---Endosomal space (Injection site & Lymph) [5]

Kdeg = THETA(6)           ; degradation rate of free drug in distribution space

VD = THETA(7)             ; volume of distribution 2

;-----FCRN recycling [5]

KOFF1 = 12.132            ; (1/h) dissociation

KON1 = 4.068/10000        ; (pM*-1h^-1) binding

KD = 29823                ; (pmol/L)

Krec = THETA(8)           ; (1/h) recycle rate of drug-FcRn complex to central comp

FcRn = 40000000           ; Total amount of FcRn (pmol/L)

FR = 0.715                ; recycling fraction of FcRn bound drug (ref)

;------GX-I7 TMDD in central [3]

Kint = THETA(9)           ; internalization of IL7R-drug complex (ref 0.642)

KOFF2 = 59.76             ; (1/h)

KON2 = 20.196/10000       ; (pM^-1 h*-1)

Rtot = THETA(10)          ; Total amount of Receptor (pmol/L)

;-----Scale

S3=VC

$DES

;------------- Injection site [1] [2]

DADT(1) = -KAIM*A(1)

DADT(2) = -KASC*A(2)

; ------Distribution1 interstitial, vascular 141

DADT(4) = KAIM*A(1) + KASC*A(2) - Kup*A(4) + Krec*A(7)*(1-FR) - K43*A(4) + K34*A(3)

;-------Distribution 2 endothelial, lymph [5]

DADT(5) = Kup*A(4) - Kdeg*A(5) - KON1*A(5)*FcRn + (KON1*(A(5)/VD) + KOFF1)*A(7)

; ------Central [3]

DADT(3) = K43*A(4) - K34*A(3) + Krec*A(7)*FR - KEL*A(3) - KON2*A(3)*Rtot + (KON2*(A(3)/VC) + KOFF2)*A(6)

; ----- CPX[6]

DADT(6) = KON2*A(3)*Rtot - (KON2*(A(3)/VC) + KOFF2 + Kint)*A(6)

DADT(7) = KON1*A(5)*FcRn - (KON1*(A(5)/VD) + KOFF1 + Krec)*A(7)

$ERROR

Cf = A(3)/VC ; (pmol/L) : total GX-17

IPRED = BASE + Cf ; add baseline IL7 conc

W = SQRT(THETA(11)**2 * IPRED**2 + THETA(12)**2)

Y = IPRED + W*EPS(1)

IRES = IPRED - DV

IWRES = IRES/W

$THETA  (0,5.26) ; 1.CL L/h

 (0,1.96) ; 2.VC L

 (0,1.22) ; 3.KA IM 1/h

 (0,0.667) ; 4.KA2 SC 1/h

 (0.01,1.21) ; 5.Q

 (0,0.642) FIX ; 6.Kdeg (ref)

 (0.0001,31.4) ; 7.VD

 (0.0001,0.000608) ; 8.Krec

 (0,0.642) FIX ; 9.Kint 1/h (ref)

 (0,1060,1370) FIX ; 10.Rtot pmol/L (T1D ref 1.37 nmol ~ healthy 1061 pmol/L)

 (0,0.332,5) ; 11.Prop

 (0,0.00303,10) ; 12.Add

 (0.1,338) ; 13.VIIM

 (0.1,956) ; 14.VISC

$OMEGA  0.292  ;        1.Q

$SIGMA  1  FIX

$COVARIANCE MATRIX=R PRINT=E

$ESTIMATION MAXEVAL=9999 PRINT=5 METHOD=1 INTER NOABORT NSIG=3 SIGL=6

$TABLE ID TIME CL VC KAIM KASC Q Kdeg Krec VIIM VISC AMT NOPRINT

ONEHEADER FILE=patab009

$TABLE ID TIME DV IPRED IWRES PRED CWRES NOPRINT ONEHEADER

FILE=sdtab009

; AMT: GX-I7 pmol x 2 = IL7 pmol, DV: pmol/L(IL-7), COHORT: 1=SC20 2=SC60 3=IM60 ug/kg

; MW of GX-17 104 kDa, IL-7 part of GX-I 20.5 kDa

(f) Dataset for case 2 (*rhil-7-hyfc*) was attached as an electrical file named by S2 Table. Dataset for case 2 (rhil-7-hyfc).csv, and the mTMDD model for case 2 (*rhil-7- hyfc*) is as follows:

$PROBLEM    GX-I7 FCRN TMDD PK

;; 1. Based on: run004_retry12

;; 2. Description: mtmdd

;; x1. Author: hyeseon

$INPUT      ID TIME AMT DV MDV COHORT CMT BASE

$DATA      S2 Table. Dataset for case 2 (rhil-7-hyfc).csv IGNORE=#

$SUBROUTINE ADVAN13 TOL=6

$MODEL      COMP(IMDEPOT) COMP(SCDEPOT) COMP(CENTRAL)

            COMP(DISTRIBUTION1) ; Absorption site Interstitial & Vascular

            COMP(DISTRIBUTION2) ; FcRn recycling space

$PK

F1 = 2

F2 = 2

;--- Central compartment [3]

CL = THETA(1) ; Cl central

VC = THETA(2) ; Vd central

KEL = CL/VC

;--- Injection depot [1] [2]

KAIM = THETA(3) ; K Absorption IM

KASC = THETA(4) ; K Absorption SC

;--- Interstitial& Vascular [4]

Kup = 0.00952              ; uptake rate to endosomal space (ref)

IF(TIME.EQ.0) Kup=0

Q = THETA(5) * EXP(ETA(1)) ; Intercompartmental clearance

;for IM

VIIM = THETA(13)           ; Volume of distribution 1 of interstitial&vascular

;for SC

VISC = THETA(14)

VI=VISC

IF(COHORT.EQ.3) VI=VIIM

K43 = Q/VI

K34 = Q/VC

;---Endosomal space (Injection site & Lymph) [5]

Kdeg = THETA(6)           ; degradation rate of free drug in distribution space

VD = THETA(7)             ; volume of distribution 2

;-----FCRN recycling [5]

KOFF1 = 12.132            ; (1/h) dissociation

KON1 = 4.068/10000        ; (pM*-1h^-1) binding

KD = 29823                ; (pmol/L)

Krec = THETA(8)           ; (1/h) recycle rate of drug-FcRn complex to central comp

FcRn = 40000000           ; Total amount of FcRn (pmol/L)

KSS1 = KD + Krec/KON1     ; (pmol/L) ; KSS1=(KOFF1 + Krec)

FR = 0.715                ; recycling fraction of FcRn bound drug (ref)

;------GX-I7 TMDD in central [3]

Kint = THETA(9)           ; internalization of IL7R-drug complex (ref 0.642)

KOFF2 = 59.76             ; (1/h)

KON2 = 20.196/10000       ; (pM^-1 h*-1)

KSS2 = (Kint + KOFF2) / KON2 ;(pM)

Rtot = THETA(10)          ; Total amount of Receptor

;-----Scale

S3=VC

$DES

;------------- Injection site [1] [2]

DADT(1) = -KAIM*A(1)

DADT(2) = -KASC*A(2)

; ------------ Distribution 2 space: QSS approximation for FcRn interaction

Afree = A(5)/VD          ; (pmol/L)

Acpx = FcRn*Afree/(KSS1+Afree)

; ------------- QSS approximation for IL7R interaction

Cfree = A(3)/VC

Ccpx = Rtot*Cfree/(KSS2+Cfree)

; ------Distribution1 interstitial, vascular 141

DADT(4) = KAIM*A(1) + KASC*A(2) - Kup*A(4) + Krec*Acpx*(1-FR)*VD - K43*A(4) + K34*A(3)

;-------Distribution 2 endothelial, lymph [5]

DADT(5) = Kup*A(4) - Krec*Acpx*VD - Kdeg*A(5)

; ------Central [3]

DADT(3) = K43*A(4) - K34*A(3) + Krec*Acpx*FR*VD - KEL*A(3) - Kint*Ccpx*VC

$ERROR

Cf = A(3)/VC ; (pmol/L) : total GX-17

IPRED = BASE + Cf ; add baseline IL7 conc

W = SQRT(THETA(11)**2 * IPRED**2 + THETA(12)**2)

Y = IPRED + W*EPS(1)

IRES = IPRED - DV

IWRES = IRES/W

$THETA

(0, 16.8)   ;    1.CL L/h

(0.0001,    3.97)   ;    2.VC L

(0, 0.848)  ;    3.KA IM 1/h

(0, 0.818)  ;    4.KA2 SC 1/h

(0.01,  73.5)   ;    5.Q

(0, 0.642) FIX ;     6.Kdeg ref

(0.0001,    0.00811)    ;    7.VD

(0.0001,    4.69)   ;    8.Krec

(0, 0.642) FIX  ;    9.Kint 1/h ref

(0, 1060) FIX   ;    10.Rtot pmol/L T1D ref 1.37 nmol ~ healthy 1061 pmol/L

(0, 0.33)   ;    11.Prop

(0, 1.14)   ;    12.Add

(0.1, 1160) ;    13.VIIM

(0.1, 8930) ;    14.VISC

$OMEGA  13.3  ;        1.Q

$SIGMA  1  FIX

$COVARIANCE MATRIX=R PRINT=E

$ESTIMATION MAXEVAL=9999 PRINT=5 METHOD=1 INTER NOABORT NSIG=3 SIGL=6

$TABLE ID TIME CL VC KAIM KASC Q Kdeg VD Krec VIIM VISC AMT

NOPRINT ONEHEADER FILE=patab012

$TABLE ID TIME DV IPRED IWRES PRED CWRES NOPRINT ONEHEADER

FILE=sdtab012

(g) Dataset for case 2 (*rhil-7-hyfc*) was attached as an electrical file named by S2 Table. Dataset for case 2 (rhil-7-hyfc).csv, and the qTMDD model for case 2 (*rhil-7- hyfc*) is as follows:

$PROBLEM    GX-I7 FCRN TMDD PK

;; 1. Based on: run001_retry16

;; 2. Description: qtmdd

;; x1. Author: hyeseon

$INPUT      ID TIME AMT DV MDV COHORT CMT BASE

$DATA      S2 Table. Dataset for case 2 (rhil-7-hyfc).csv IGNORE=#

$SUBROUTINE ADVAN13 TOL=6

$MODEL      COMP(IMDEPOT) COMP(SCDEPOT) COMP(CENTRAL)

            COMP(DISTRIBUTION1) ; Absorption site Interstitial & Vascular

            COMP(DISTRIBUTION2) ; FcRn recycling space

$PK

F1 = 2

F2 = 2

;--- Central compartment [3]

CL = THETA(1) ; Cl central

VC = THETA(2) ; Vd central

KEL = CL/VC

;--- Injection depot [1] [2]

KAIM = THETA(3) ; K Absorption IM

KASC = THETA(4) ; K Absorption SC

;--- Interstitial& Vascular [4]

Kup = 0.00952              ; uptake rate to endosomal space (ref)

IF(TIME.EQ.0) Kup=0

Q = THETA(5) * EXP(ETA(1)) ; Intercompartmental clearance

;for IM

VIIM = THETA(13)           ; Volume of distribution 1 of interstitial&vascular

;for SC

VISC = THETA(14)

VI=VISC

IF(COHORT.EQ.3) VI=VIIM

K43 = Q/VI

K34 = Q/VC

;---Endosomal space (Injection site & Lymph) [5]

Kdeg = THETA(6)           ; degradation rate of free drug in distribution space

VD = THETA(7)             ; volume of distribution 2

;-----FCRN recycling [5]

KOFF1 = 12.132            ; (1/h) dissociation

KON1 = 4.068/10000        ; (pM*-1h^-1) binding

KD = 29823                ; (pmol/L)

Krec = THETA(8)           ; (1/h) recycle rate of drug-FcRn complex to central comp

FcRn = 40000000           ; Total amount of FcRn (pmol/L)

KSS1 = KD + Krec/KON1     ; (pmol/L) ; KSS1=(KOFF1 + Krec)

FR = 0.715                ; recycling fraction of FcRn bound drug (ref)

;------GX-I7 TMDD in central [3]

Kint = THETA(9)           ; internalization of IL7R-drug complex (ref 0.642)

KOFF2 = 59.76             ; (1/h)

KON2 = 20.196/10000       ; (pM^-1 h*-1)

KSS2 = (Kint + KOFF2) / KON2 ;(pM)

Rtot = THETA(10)          ; Total amount of Receptor

;-----Scale

S3=VC

$DES

;------------- Injection site [1] [2]

DADT(1) = -KAIM*A(1)

DADT(2) = -KASC*A(2)

; ------------ Distribution 2 space: QSS approximation for FcRn interaction

Atot = A(5)/VD          ; (pmol/L)

DF = Atot - FcRn - KSS1 ; (pmol/L)

Afree = 0.5 * (DF + SQRT(DF**2 + 4*KSS1*Atot)) ; AMT of active free drug (pmol/L) in distribution space derived from total amount in distribution space

                                           ; derived from total amount in distribution space

Acpx = (FcRn*Afree)/(KSS1 + Afree)

; ------------- QSS approximation for IL7R interaction

Ctot = A(3)/VC          ;(pmol/L) ; total GX-17

D = Ctot - Rtot - KSS2  ;(pmol/L)

Cfree = 0.5*(D + SQRT(D**2 + 4*KSS2*Ctot))     ; AMOUNT of free GX-17 (pmol/L)

                                           ; derived from total conc of GX-17

Ccpx = (Rtot*Cfree)/(KSS2 + Cfree) ;GX-I7-IL7R complex

; ------Distribution1 interstital, vascular 141

DADT(4) = KAIM*A(1) + KASC*A(2) - Kup*A(4) + Krec*Acpx*(1-FR)*VD - K43*A(4) + K34*Cfree*VC

;-------Distribution 2 endothelial, lymph [5]

DADT(5) = Kup*A(4) - Krec*Acpx*FR*VD - Krec*Acpx*(1-FR)*VD - Kdeg*Afree*VD

; ------Central [3]

DADT(3) = K43*A(4) - K34*Cfree*VC + Krec*Acpx*FR*VD - KEL*Cfree*VC - Kint*(Ccpx)*VC

$ERROR

Ct = A(3)/VC ; (pmol/L) : total GX-17

DD = Ct - Rtot - KSS2 ; (pmol/L)

Cf = 0.5 * (DD + SQRT(DD**2 + 4*KSS2*Ct)) ; (pmol/L) : free GX-17

IPRED = BASE + Cf ; add baseline IL7 conc

W = SQRT(THETA(11)**2 * IPRED**2 + THETA(12)**2)

Y = IPRED + W*EPS(1)

IRES = IPRED - DV

IWRES = IRES/W

$THETA  (0,5.22) ; 1.CL L/h

 (0,1.98) ; 2.VC L

 (0,1.23) ; 3.KA IM 1/h

 (0,0.672) ; 4.KA2 SC 1/h

 (0.01,1.21) ; 5.Q

 (0,0.642) FIX ; 6.Kdeg (ref)

 (0.0001,28.1) ; 7.VD

 (0.0001,0.000603) ; 8.Krec

 (0,0.642) FIX ; 9.Kint 1/h (ref)

 (0,1060,1370) FIX ; 10.Rtot pmol/L (T1D ref 1.37 nmol ~ healthy 1061 pmol/L)

 (0,0.332,5) ; 11.Prop

 (0,0.00348,10) ; 12.Add

 (0.1,340) ; 13.VIIM

 (0.1,962) ; 14.VISC

$OMEGA  0.295  ;        1.Q

$SIGMA  1  FIX

$COVARIANCE MATRIX=R PRINT=E

$ESTIMATION MAXEVAL=9999 PRINT=5 METHOD=1 INTER NOABORT NSIG=3 SIGL=6

$TABLE ID TIME CL VC KAIM KASC Q Kdeg VD Krec VIIM VISC AMT

NOPRINT ONEHEADER FILE=patab016

$TABLE ID TIME DV IPRED IWRES PRED CWRES NOPRINT ONEHEADER

FILE=sdtab016

(h) Dataset for case 2 (*rhil-7-hyfc*) was attached as an electrical file named by S2 Table. Dataset for case 2 (rhil-7-hyfc).csv, and the pTMDD model for case 2 (*rhil-7- hyfc*) is as follows:

$PROBLEM    GX-I7 FCRN TMDD PK

;; 1. Based on: run010_retry16

;; 2. Description: ttmdd-2

;; x1. Author: hyeseon

$INPUT      ID TIME AMT DV MDV COHORT CMT BASE

$DATA      S2 Table. Dataset for case 2 (rhil-7-hyfc).csv IGNORE=#

$SUBROUTINE ADVAN13 TOL=6

$MODEL      COMP(IMDEPOT) COMP(SCDEPOT) COMP(CENTRAL)

            COMP(DISTRIBUTION1) ; Absorption site Interstitial & Vascular

            COMP(DISTRIBUTION2) ; FcRn recycling space

$PK

F1 = 2

F2 = 2

;--- Central compartment [3]

CL = THETA(1) ; Cl central

VC = THETA(2) ; Vd central

KEL = CL/VC

;--- Injection depot [1] [2]

KAIM = THETA(3) ; K Absorption IM

KASC = THETA(4) ; K Absorption SC

;--- Interstitial& Vascular [4]

Kup = 0.00952              ; uptake rate to endosomal space (ref)

IF(TIME.EQ.0) Kup=0

Q = THETA(5) * EXP(ETA(1)) ; Intercompartmental clearance

;for IM

VIIM = THETA(13)           ; Volume of distribution 1 of interstitial&vascular

;for SC

VISC = THETA(14)

VI=VISC

IF(COHORT.EQ.3) VI=VIIM

K43 = Q/VI

K34 = Q/VC

;---Endosomal space (Injection site & Lymph) [5]

Kdeg = THETA(6)           ; degradation rate of free drug in distribution space

VD = THETA(7)             ; volume of distribution 2

;-----FCRN recycling [5]

KOFF1 = 12.132            ; (1/h) dissociation

KON1 = 4.068/10000        ; (pM*-1h^-1) binding

KD = 29823                ; (pmol/L)

Krec = THETA(8)           ; (1/h) recycle rate of drug-FcRn complex to central comp

FcRn = 40000000           ; Total amount of FcRn (pmol/L)

KSS1 = KD + Krec/KON1     ; (pmol/L) ; KSS1=(KOFF1 + Krec)

FR = 0.715                ; recycling fraction of FcRn bound drug (ref)

;------GX-I7 TMDD in central [3]

Kint = THETA(9)           ; internalization of IL7R-drug complex (ref 0.642)

KOFF2 = 59.76             ; (1/h)

KON2 = 20.196/10000       ; (pM^-1 h*-1)

KSS2 = (Kint + KOFF2) / KON2 ;(pM)

Rtot = THETA(10)          ; Total amount of Receptor

;-----Scale

S3=VC

$DES

;------------- Injection site [1] [2]

DADT(1) = -KAIM*A(1)

DADT(2) = -KASC*A(2)

; ------------ Distribution 2 space: QSS approximation for FcRn interaction

Atot = A(5)/VD          ; (pmol/L)

Acpx = (FcRn*Atot)/(KSS1 + Atot + FcRn)

Afree = Atot-Acpx

; ------------- QSS approximation for IL7R interaction

Ctot = A(3)/VC          ;(pmol/L) ; total GX-17

Ccpx = (Rtot*Ctot)/(KSS2 + Ctot + Rtot) ;GX-I7-IL7R complex

Cfree = Ctot-Ccpx

; ------Distribution1 interstital, vascular 141

DADT(4) = KAIM*A(1) + KASC*A(2) - Kup*A(4) + Krec*Acpx*(1-FR)*VD - K43*A(4) + K34*Cfree*VC

;-------Distribution 2 endothelial, lymph [5]

DADT(5) = Kup*A(4) - Krec*Acpx*FR*VD - Krec*Acpx*(1-FR)*VD - Kdeg*Afree*VD

; ------Central [3]

DADT(3) = K43*A(4) - K34*Cfree*VC + Krec*Acpx*FR*VD - KEL*Cfree*VC - Kint*(Ccpx)*VC

$ERROR

Ct = A(3)/VC ; (pmol/L) : total GX-17

Ccp = (Rtot*Ct)/(KSS2 + Ct + Rtot) ;GX-I7-IL7R complex

Cf= Ct-Ccp

IPRED = BASE + Cf ; add baseline IL7 conc

W = SQRT(THETA(11)**2 * IPRED**2 + THETA(12)**2)

Y = IPRED + W*EPS(1)

IRES = IPRED - DV

IWRES = IRES/W

$THETA  (0,5.27) ; 1.CL L/h

 (0,2.08) ; 2.VC L

 (0,1.24) ; 3.KA IM 1/h

 (0,0.679) ; 4.KA2 SC 1/h

 (0.01,1.3) ; 5.Q

 (0,0.642) FIX ; 6.Kdeg (ref)

 (0.0001,35.9) ; 7.VD

 (0.0001,0.000621) ; 8.Krec

 (0,0.642) FIX ; 9.Kint 1/h (ref)

 (0,1060,1370) FIX ; 10.Rtot pmol/L (T1D ref 1.37 nmol ~ healthy 1061 pmol/L)

 (0,0.332,5) ; 11.Prop

 (0,0.00486,10) ; 12.Add

 (0.1,358) ; 13.VIIM

 (0.1,1020) ; 14.VISC

$OMEGA  0.302  ;        1.Q

$SIGMA  1  FIX

$COVARIANCE MATRIX=R PRINT=E

$ESTIMATION MAXEVAL=9999 PRINT=5 METHOD=1 INTER NOABORT NSIG=3 SIGL=6

$TABLE ID TIME CL VC KAIM KASC Q Kdeg VD Krec VIIM VISC AMT

NOPRINT ONEHEADER FILE=patab016

$TABLE ID TIME DV IPRED IWRES PRED CWRES NOPRINT ONEHEADER

FILE=sdtab016
