# Supplementary figures and images for: Validity conditions of approximations for a target-mediated drug disposition model: A novel first-order approximation and its comparison to other approximations
Source: PLoS Comput Biol. 2024 Apr 24;20(4):e1012066. doi: 10.1371/journal.pcbi.1012066 (PMC11090311; doi:10.1371/journal.pcbi.1012066)

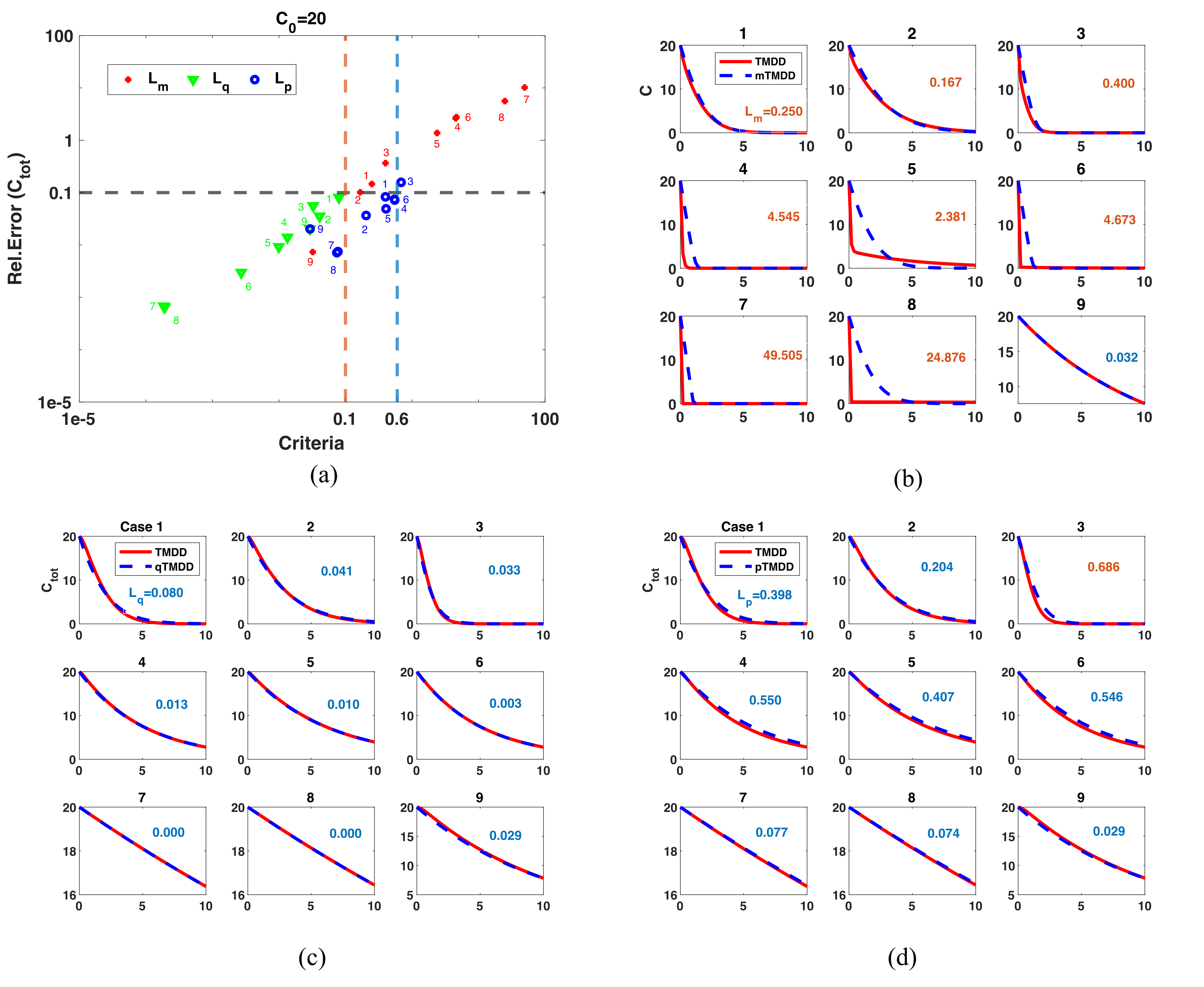

Supplement: S1 Fig — (a) Relative errors of mTMDD, qTMDD, and pTMDD are small when Lm, Lq, and Lp are small, respectively. The number in the figure represents the case number in Table 2. Here, 20 units of initial drug were used. (b) mTMDD accurately approximated TMDD when Lm<0.1 (blue font) but failed otherwise (red font). The numbers in the figure represent the value of Lm. Note that C represents Ctot in mTMDD because it assumes RC is negligible. (c) qTMDD accurately approximated TMDD for all cases because Lq<0.6. (d) pTMDD accurately approximated TMDD for the total drug when Lp<0.6 (blue font) but failed otherwise (red font). (TIF) [file pcbi.1012066.s004.tif]

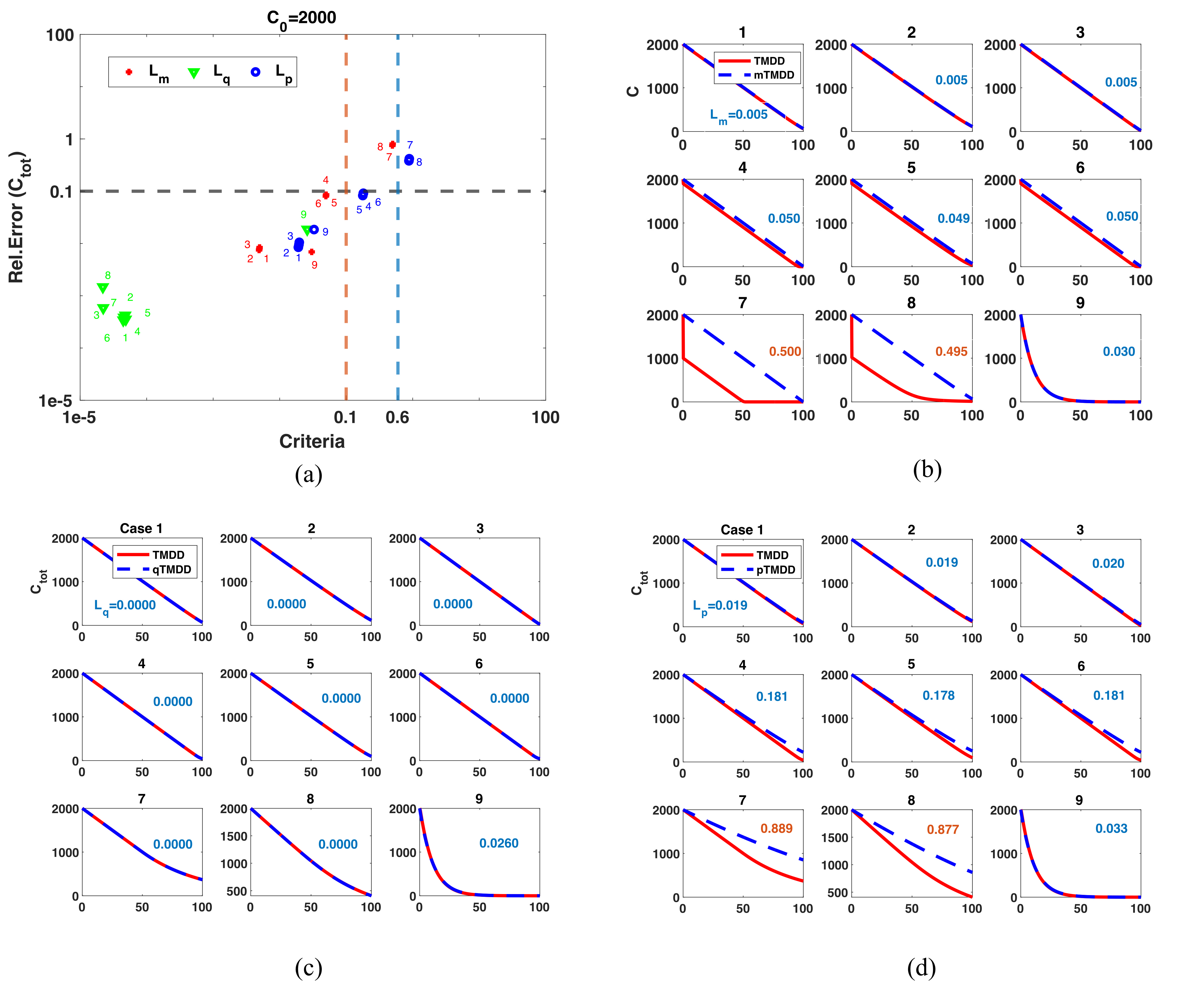

Supplement: S2 Fig — (a) Relative errors of mTMDD, qTMDD, and pTMDD are small when Lm, Lq, awpnd Lp are small, respectively. The number in the figure represents the case number in Table 2. Here, 2000 units of initial drug were used. (b) mTMDD accurately approximated TMDD when Lm<0.1 (blue font) but failed otherwise (red font). The numbers in the figure represent the value of Lm. Note that C represents Ctot in mTMDD because it assumes RC is negligible. (c) qTMDD accurately approximated TMDD for all cases because Lq<0.6. (d) pTMDD accurately approximated TMDD for the total drug when Lp<0.6 (blue font) but failed otherwise (red font). (TIF) [file pcbi.1012066.s005.tif]

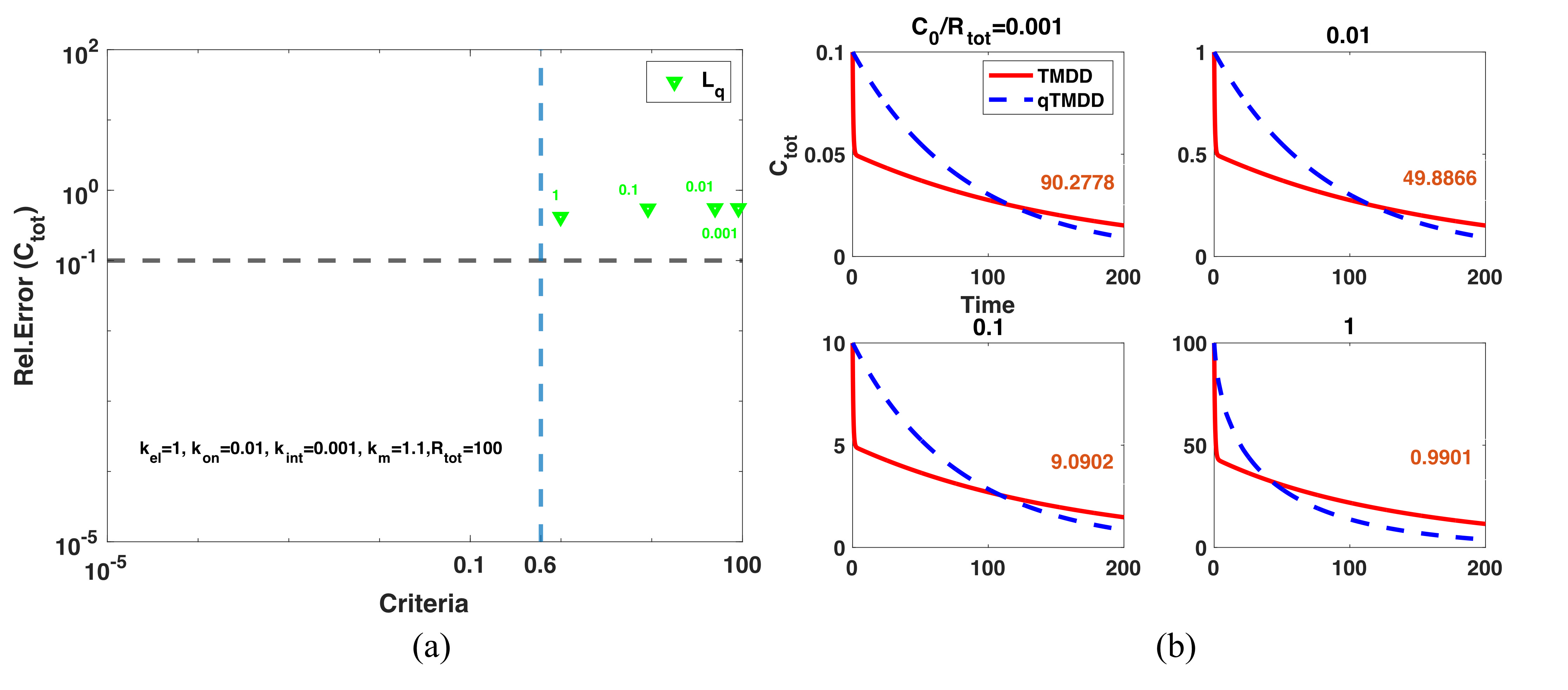

Supplement: S3 Fig — (a) We used the values of parameters from Case 9 of Table 2 except for Rtot and kel. We used Rtot = 100 and kel = 1. Furthermore, we varied initial drug concentrations (C0) as 0.1, 1, 10 and 100 units so that C0/Rtot changes. C0/Rtot is represented as the numbers in the figure. In all these cases, kel≪kon(km+C0) is not satisfied because kel = 1 and the values of kon(km+C0) are 0.012 (C0/Rtot = 0.001), 0.021 (0.01), 0.111 (0.1) and 1.011 (1). As a result, Lq exceeded 0.6 regardless C0/Rtot, resulting in relative errors of qTMDD greater than 0.1. (b) Since Lq>0.6 (red font), qTMDD fails to approximate TMDD regardless of C0/Rtot. (TIF) [file pcbi.1012066.s006.tif]
