# Supplementary material for: Validity conditions of approximations for a target-mediated drug disposition model: A novel first-order approximation and its comparison to other approximations
Source: PLoS Comput Biol. 2024 Apr 24;20(4):e1012066. doi: 10.1371/journal.pcbi.1012066 (PMC11090311; doi:10.1371/journal.pcbi.1012066)
Supplement: Attachment — Submitted filename: review response_v1_final.docx [file pcbi.1012066.s007.docx]

Review response for “**Validity Conditions of Approximations for a Target-Mediated Drug Disposition Model: A Novel First-Order Approximation and Its Comparison to Other Approximations”**

We thank the editor and the reviewers for their valuable feedback on our manuscript, constructive comments, and suggestions. In response to the reviewers’ comments and guidance, we have significantly edited our manuscript. Below, we give our detailed responses to the comments and describe the changes in the manuscript. Our responses are denoted in blue, while the revised portions of the manuscript are highlighted in green.

Major comments

**Reviewer #1:**

1) This is an interesting and novel investigation of the important biological equations that are often used in drug development. I do not have any major comments to what was done in the manuscript. However, to make it more useful, it would be helpful to consider a more general case of a two compartment system (all monoclonal antibodies have bi-exponential kinetic) and remove the restriction kint=kdeg Otherwise, application of the work to real-life cases will be very limited. With that, I can recommend the manuscript to for the publication.

Thanks for pointing this out. Indeed, $k_{deg}=k_{int}$ has been commonly assumed to derive the various approximations of TMDD models previously [1-3]. As the goal of this study was to provide the validity conditions for these models, assuming $k_{deg}=k_{int}$ was required so that the total target concentration $R_{tot}$ was constant.

Nevertheless, to show the utility of the simplified models (mTMDD, qTMDD, and pTMDD) based on the assumption of $k_{int}=k_{deg}$, we performed additional PK parameter estimations with real data. Specifically, we applied the simplified models to antibody-drug conjugate real-world data based on a physiologically based mechanistic FcRn model for antibody coupled with TMDD [4]. Indeed, the simplified TMDD models could accurately estimate PK parameters even with the assumption (see our response to comment 6 of reviewer #2).

Furthermore, we agree with the reviewer that extending the current framework to the more general case is critical. Thus, we have discussed this point in the discussion as follows:

*Discussion:*

We assumed that the total target concentrations were constant (i.e., $k_{int}=k_{deg})$ in order to prevent the validity criteria complex. While this assumption is commonly used [1-3], developing more flexible criteria without the assumption may offer a comprehensive assessment of the TMDD model, facilitating better-informed decisions. We also assumed that the drug amount in the peripheral compartment was zero. Without this assumption, the peripheral compartment needs to be incorporated in Eq. (1). Future work will investigate a new approach for validity criteria in generalizing the TMDD model considering a non-zero drug amount in the peripheral compartment.

**Reviewer #2:**

1) While the work presented in this manuscript have merit and relevance to the development of drugs demonstrating TMDD, the presentation of the work is not lucid, and the author loses the important points being made to the reader in the forest of math. Below are a few suggestions to majorly modify the manuscript. From the title itself the author should mention that they are proposing a new approximation of TMDD: something like “A Novel First-Order Approximation of TMDD Model and its Comparison to Other Approximations”. Thus, the reader knows exactly what they are reading.

We agree with the reviewer. Thus, we have considerably revised the manuscript to enhance the readership. Furthermore, we have also revised the title to "Validity Conditions of Approximations for a Target-Mediated Drug Disposition Model: A Novel First-Order Approximation and Its Comparison to Other Approximations**”** as the reviewer suggested.

2) While all the math presented in the paper is needed for the experts to verify the work, maybe a lot can be shuttled to supplementary information so author can focus on the performance of their approximation with other approximations.

We agree with the reviewer. Now, we have revised the results section to include minimal mathematics and have moved the complex mathematical part to the materials and methods section. Thus, the manuscript has been greatly improved to have wider readership thanks to the suggestion from the reviewer.

3) The authors need to show the PK data used by them and then quality of fit by each model (by superimposing fits over the observed data). In the absence of such figures - it is hard to assess which model is off by how much. Also, the figures and performance of different approximations is hard to grasp, which may suggest making different/better figures may be of help.

We have now completely revised the figure to clearly show the error of each model (Fig. 1-3) according to the reviewer’s suggestions as follows:

**Figure 1. When** $\boldsymbol{L}_{\boldsymbol{m}}\boldsymbol{,}\boldsymbol{L}_{\boldsymbol{q}}\boldsymbol{,}\mathbf{and} \boldsymbol{L}_{\boldsymbol{p}}$ **are small, mTMDD, qTMDD, and pTMDD provide accurate approximation for TMDD.** (a) Relative errors of mTMDD, qTMDD, and pTMDD are small when $L_{m}, L_{q}, \mathrm{and} L_{p}$ are small, respectively. The number in the figure represents the case number in Table 2. Here, 200 units of initial drug were used. For 20 and 2000 units of initial drugs, see Figs. S2-S3. (b) mTMDD accurately approximated TMDD when $L_{m}<0.1$ (blue font) but failed otherwise (red font). The numbers in the figure represent the value of $L_{m}$. Note that C represents $C_{tot}$ in mTMDD because it assumes RC is negligible. (c) qTMDD accurately approximated TMDD for all cases because $L_{q}<0.6$. (d) pTMDD accurately approximated TMDD for the total drug when $L_{p}<0.6$(blue font) but failed otherwise (red font).

**Figure 2. The accuracy of the models strongly depends on the ratio between drug concentration and total receptor concentration.** (a) Relative errors are plotted against validity criteria for various drug concentrations with the parameter set obtained from [5]. The parameter values of $k_{el}, k_{on}, k_{off}, k_{int,}$ and $R_{tot}$ were 0.05, 0.5, 0.1, 1, 20, respectively, so that $k_{m}=2.2$. The number shown in the figure represents the value of $C_{0}/R_{tot}$. (b) The relative errors of the models with respect to $C_{0}/R_{tot}.$ mTMDD model was accurate only when $C_{0}/R_{tot}$ was large. The pTMDD model was accurate except for $C_{0}/R_{tot}\approx1$. The qTMDD model was accurate regardless of $C_{0}/R_{tot}$. (c) pTMDD and qTMDD, but not mTMDD were accurate when $C_{0}/R_{tot}=0.1.$ (d) When $C_{0}/R_{tot}=1$, only qTMDD was accurate. (e-f) As $C_{0}/R_{tot}$ increased, all models became more accurate.

Two antibody-drug conjugate clinical trial studies were selected from real case application studies. Case 1 was *hIL-1Ra-hyFc* (human interleukin-1 receptor antagonist components into one antibody-derived fragment crystallizable portion) for the case of large $C_{0}/R_{tot}$(=22.33) and case 2 was *rhIL-7-hyFc* (recombinant human interleukin-7, hybrid Fc-fused) for the case of small $C_{0}/R_{tot}$(=0.01), respectively. The detailed trial information of both studies was referred to by *Ngo. et al.,* [6] and *Lee et al.* [7]. Both were semi-mechanistic models considering TMDD and FcRn-mediated recycling, and few details were modified. We applied the three methods (mTMDD, qTMDD, and pTMDD) to the binding of a drug to its target in consideration of biological processes, and other processes were described as first-order kinetics.

Parameter optimization for the models was performed using the first-order conditional estimation with interaction (FOCE-I) method using NONMEM 7.5 and PsN 5.3.1 software, and model performance was evaluated by model diagnostics criteria and diagnostic plots.

**Figure 3. Visual predictive checks of (A) Case 1 for *hIL-1Ra-hyFc*** **(**$\boldsymbol{C}_{\boldsymbol{0}}\boldsymbol{/}\boldsymbol{R}_{\boldsymbol{tot}}\boldsymbol{=22.33}$) **and (B) Case 2 for *rhIL-7-hyFc*** **(**$\boldsymbol{C}_{\boldsymbol{0}}\boldsymbol{/}\boldsymbol{R}_{\boldsymbol{tot}}\boldsymbol{=}\mathbf{0.01}$) **[4]**. The red line indicates the 50th percentile of observations, and the lower and upper blue lines indicate the 5th and 95th percentile of observations, respectively. Blue shades indicate model-predicted confidence intervals of the 5th and 95th percentile, and red shade indicates model-predicted confidence intervals of the 50th percentile. For all PK models of case 1 and the TMDD, qTMDD, and pTMDD PK models of case 2, the model-predicted confidence interval encompassed 5th percentile, 95th percentile, and median observations. Hence, the approximation models reasonably explained the observation data. For the mTMDD PK model of case 2, the 95th percentile of the observations was low compared to the 95% confidence interval. Therefore, the model cannot be said to predict appropriately.

4) All the “conditions” in which one model works vs. other is dizzying. Especially because all of them have some symbols less than or higher than other symbols. The author should try to summarize the conditions in a physiological/pharmacological sense so the reader can easily understand the caveats for using each equation.

Now, we have revised the results section so that the conditions can be understood in a physiological/pharmacological sense with minimal mathematical symbols. Furthermore, we have also provided simpler criteria which can be understood without any mathematics (Table 3).

5) In Table 2, some cases have Koff=0. This is impossible and not relevant for TMDD models. Please confine your analysis to parameter ranges that are realistic.

We completely agree with the reviewer that $k_{off}=0$ is unrealistic, and the original intention was to describe the condition when the drug was slowly dissociated from the target. Thus, we have changed it to $k_{off}=0.01$ as the very small value for representing slow dissociation of drugs from targets in cases 1,4, and 7 of Table 2. Despite this change, the results are nearly the same.

6) Eventually it turns out the new approximation proposed by the author is no better than existing qTMDD approximation. Thus, the authors owe the reader an explanation as to why would one still pursue the new approximation, especially since it can be applied to conditions that are lesser than the one allowed by qTMDD. Saying, “qTMDD may have higher run time” is not good enough. Please do some actual estimations and report the run times and prove the point using real data than an expectation.

Thanks for the suggestion. Now, we have compared the fitting performance of PK models with mTMDD, qTMDD, and pTMDD models using real data with two antibody-drug conjugate clinical trial studies. This showed that although pTMDD showed similar accuracy with qTMDD, its computation time was about two times shorter compared to qTMDD (Table 4) as follows:

**Table 4. Summary of the parameters estimated in the antibody-drug conjugate real-world data application study.**

| - | Case 1 *for hIL-1Ra-hyFc*  ($C_{0}/R_{tot}=22.33$) | | | | Case 2 *for rhIL-7-hyFc*  ($C_{0}/R_{tot}=0.01$) | | | |
| --- | --- | --- | --- | --- | --- | --- | --- | --- |
| - | TMDD | mTMDD | qTMDD | pTMDD | TMDD | mTMDD | qTMDD | pTMDD |
| *OFV* | 1556.40 | 1564.45 | 1556.59 | 1561.90 | 1552.90 | 1611.92 | 1552.90 | 1553.14 |
| *AIC* | 2194.40 | 1926.45 | 1918.59 | 1923.90 | 1842.90 | 1855.92 | 1796.90 | 1797.14 |
| *CL* | 0.208 | 0.209 | 0.207 | 0.199 | 5.27 | 16.8 | 5.23 | 5.27 |
| *Ka* | 1.22 | 1.17 | 1.21 | 1.23 | 1.22(im), 0.665(sc) | 0.848(im), 0.817(sc) | 1.23(im), 0.697(sc) | 1.24(im), 0.679(sc) |
| *Q* | 0.0284 | 0.0145 | 0.0292 | 0.0279 | 1.21 | 73.5 | 1.21 | 1.3 |
| *Vc* | 11.4 | 11.6 | 11.2 | 11.3 | 1.95 | 3.96 | 1.97 | 2.07 |
| *Vd* | 117 | - | - | - | 34.8 | 0.00810 | 28.2 | 35.9 |
| Elapsed time | 3763.88 | 403.25 | 1113.53 | 555.99 | 87.87 | 44.35 | 65.26 | 39.11 |

*OFV*: objective function value, *AIC*: Akaike information criterion, *CL*: clearance, *Ka*: absorption rate constant, *Vc*: volume of distribution (central), *Vd*: volume of distribution (peripheral). A few more parameters related to TMDD, such as *K_deg_* (degradation rate constant), *K_on_* (binding rate of drug and receptor), *K_off_* (dissociation rate constant of drug and receptor), *K_uptake_* (uptake rate of antibody), and *K_recycle_* (recycling rate constant from distribution space to a central compartment) were also used for the PK models, but are not shown in the table.

7) Lastly, the author need to read a bit more about the history of TMDD. The first time this was proposed by Levy (PMID: 7924119), and it does not just change metabolism of the drug, it can also change distribution. Maybe the author can benefit from collaboration with a PK/PD Modeling and Simulation expert in the field of protein therapeutics.

Thanks for the suggestion. Now, we have provided more details about the history of TMDD in the introduction as follows:

*Drugs that bind to their target sites can undergo a nonlinear pharmacokinetic phenomenon that is called “target-mediated drug disposition” (TMDD), first introduced by Levy [8-12]. TMDD occurs when the binding of a drug to its target influences the distribution and elimination of the drug, and is particularly common with biologics, such as monoclonal antibodies [13]*. TMDD can be described with a system of ordinary differential equations for a drug, target, and drug-target complex [14]. The TMDD model takes into account the fact that drugs can bind to their target molecules and be eliminated from the body, or they can dissociate from their target molecules and re-enter the circulation. The model is critical to drug development for predicting drug efficacy and safety, selecting drug candidates, and optimizing lead compounds [10, 15-17].

*The TMDD model can be simplified to increase computational efficiency, make analysis easier, and provide insights into the behavior of the system [18-20]*. Furthermore, it is critical to avoid over-parameterization and to simplify the complexity of the TMDD model [5]. This is particularly important when dealing with relatively sparse clinical data, because of ethical issues. The application of these techniques helps to streamline the model, making it more manageable and interpretable, while ensuring robust and accurate predictions despite limited data availability. Applications generally employ the following processes: parameter reduction, which reduces the number of model parameters; state variable reduction, which reduces the number of state variables in the model; and structural model reduction, which simplifies the structure of the model [1, 16, 21].

Furthermore, as the reviewer suggested, we have collaborated with PK/PD experts and performed the PK parameter estimations with real data (Fig. 3 and Table 4).

**Reviewer #3:** The manuscript is well written and the methodology is sound. However, the are some critical concerns that needs to be addressed:

1) Target mediated drug disposition is NOT a phenomenon is which drug metabolism in influence by interaction with target molecules. What is influence is the disposition of the drug, as the term TMDD indicates, and that encompass the distribution and elimination, but not explicitly drug metabolism, specifically for monoclonal antibodies.

Thanks for the suggestion. We have revised the description of TMDD based on *Levy*'s definition in the abstract/introduction section as follows:

*Abstract***:** *Target-mediated drug disposition (TMDD) is a phenomenon characterized by a drug's high-affinity binding to a target molecule, significantly influencing its pharmacokinetic profile within the organism.*

*Introduction*: *Drugs that bind to their target sites with high affinity can undergo a nonlinear pharmacokinetic phenomenon that is called “target-mediated drug disposition” (TMDD), first introduced by Levy [8-12]. TMDD occurs when the binding of a drug to its target influences the distribution and elimination of the drug, and is particularly common with biologics, such as monoclonal antibodies [13].*

2) The approximation of the TMDD model requires in specific projects depend on the data available. For instance, if available data of free and total drug, as well as free and total target are available, the full TMDD model is identifiable and the quasi-steady-state (QSS), quasi equilibrium (QE) and Michaelis-Menten (MM) are not really needed. However, if only free drug data are available, then the approximations are needed. It would be good to incorporate this concept into the introduction of the manuscript. The simulations should cover different data availability situations, not only when only free drug concentration is available.

Thanks for the suggestion. We have added the suggested part in the introduction as follows:

The TMDD model can be simplified to increase computational efficiency, make analysis easier and provide insights into the behavior of the system [18-20]. Furthermore, it is critical to avoid over-parameterization and to simplify the complexity of the TMDD model [5]. *This is particularly important when dealing with relatively sparse clinical data, because of ethical issues.* The application of these techniques helps to streamline the model, making it more manageable and interpretable, while ensuring robust and accurate predictions despite limited data availability. Applications generally employ the following processes: parameter reduction, which reduces the number of model parameters; state variable reduction, which reduces the number of state variables in the model; and structural model reduction, which simplifies the structure of the model [1, 16, 21].

3) The situation where the target concentration exceeds drug concentration is very rare in drug development because the dose of the compounds is increased until the target is covered. Therefore, most of the dose escalation studies are conducted to achieving the maximal drug effect, consequently, the situation where target concentration exceeds drug concentration is a theoretical concern of limited relevance, given that is very rare to find a situation like this in drug development. As a matter of fact, it would be good that the authors present a case study where this situation become relevant and list the clinical situations where this approach might be helpful. Please note paclitaxel does not exhibit TMDD but the nonlinear PK behavior is due to the liposome formulation.

Thanks for the suggestion. The paclitaxel example was removed since we agreed with reviewer's comments. We have now discussed the case when the drug concentration is lower than the target concentration as follows in the introduction and results:

*Introduction:*

Previous studies have compared the advantages and disadvantages of various TMDD models: the quasi equilibrium (QE) model reduced with quasi-equilibrium, the Michaelis-Menten (MM) model reduced with standard quasi-steady state approximation (sQSSA), and the OSSA model reduced with total QSSA (tQSSA) [1, 9, 16, 22]. The QE model has proven effective under conditions of higher drug concentrations than target concentrations but has not held when the internalization rate of the complex is non-negligible. Subsequently, the MM model has been introduced, streamlining equations and parameters for more efficient outcomes [16]. Although widely applied, the MM model requires significantly higher drug concentrations than the target for validation [2, 5]. The MM model often needs dose correction for the initial condition to prevent data misfitting and biased parameter estimates [23, 24]. In addressing these challenges, *Gibianski et al.* introduced the QSSA model, which offers improved approximations across various cases [5]. While the validity conditions of approximations have been investigated, the research has been performed in limited conditions, such as excessive drug concentration over the target concentration [3, 16]. *While the condition of excessive drug concentration is common, there are cases when the target concentration is comparable to or exceeds that of the drugs, in particular for micro-dosing studies or for drugs having highly non-specific protein binding.*

*Results:*

Next, we applied the approximate models to antibody-drug conjugate real-world data based on a physiologically based mechanistic FcRn model for antibody coupled with TMDD [4]. These PK models with TMDD, mTMDD, qTMDD, and pTMDD were fitted to totals of 640 and 456 time verses plasma concentration data of case 1 for *hIL-1Ra-hyFc* and case 2 for *rhIL-7-hyFc* from randomized clinical trials, respectively.

In the case 1 data, when $C_{0}/R_{tot}$ (=22.33) was large, the PK models with mTMDD, qTMDD, and pTMDD provided a similar estimation of PK parameters with the PK model with TMDD (Table 4), supporting our simple criteria (Table 3). Furthermore, all PK models reasonably explained the observation data according to visual predictive check plots (Fig. 3a): all the model-predicted confidence intervals encompassed 5%, 95th percentile, and median observations, so the approximation models reasonably explained the observation data.

On the other hand, in case 2 data where $C_{0}/R_{tot}$ (=0.01) was small, the PK models with qTMDD and pTMDD, but not the mTMDD model, provided a similar estimation with the PK model with TMDD (Table 4). This was consistent with our simple criteria (Table 3). From the result of visual predictive check plots, the PK models with TMDD, qTMDD, and pTMDD suitably explained the observation data (Fig. 3b). However, the PK models with the mTMDD model did not explain the observation data since the 95th percentile of the observations was located too low to be adequately encompassed by the model-predicted confidence interval of 95% (Fig. 3b).

**Table 4. Summary of the parameters estimated in the antibody-drug conjugate real-world data application study.**

| - | Case 1 *for hIL-1Ra-hyFc*  ($C_{0}/R_{tot}=22.33$) | | | | Case 2 *for rhIL-7-hyFc*  ($C_{0}/R_{tot}=0.01$) | | | |
| --- | --- | --- | --- | --- | --- | --- | --- | --- |
| - | TMDD | mTMDD | qTMDD | pTMDD | TMDD | mTMDD | qTMDD | pTMDD |
| *OFV* | 1556.40 | 1564.45 | 1556.59 | 1561.90 | 1552.90 | 1611.92 | 1552.90 | 1553.14 |
| *AIC* | 2194.40 | 1926.45 | 1918.59 | 1923.90 | 1842.90 | 1855.92 | 1796.90 | 1797.14 |
| *CL* | 0.208 | 0.209 | 0.207 | 0.199 | 5.27 | 16.8 | 5.23 | 5.27 |
| *Ka* | 1.22 | 1.17 | 1.21 | 1.23 | 1.22(im), 0.665(sc) | 0.848(im), 0.817(sc) | 1.23(im), 0.697(sc) | 1.24(im), 0.679(sc) |
| *Q* | 0.0284 | 0.0145 | 0.0292 | 0.0279 | 1.21 | 73.5 | 1.21 | 1.3 |
| *Vc* | 11.4 | 11.6 | 11.2 | 11.3 | 1.95 | 3.96 | 1.97 | 2.07 |
| *Vd* | 117 | - | - | - | 34.8 | 0.00810 | 28.2 | 35.9 |
| Elapsed time | 3763.88 | 403.25 | 1113.53 | 555.99 | 87.87 | 44.35 | 65.26 | 39.11 |

*OFV*: objective function value, *AIC*: Akaike information criterion, *CL*: clearance, *Ka*: absorption rate constant, *Vc*: volume of distribution (central), *Vd*: volume of distribution (peripheral). Few more parameters related to TMDD, such as *K_deg_* (degradation rate constant), *K_on_* (binding rate of drug and receptor), *K_off_* (dissociation rate constant of drug and receptor), *K_uptake_* (uptake rate of antibody), and *K_recycle_* (recycling rate constant from distribution space to a central compartment) were also used for the PK models, but are not shown in the table.

4) Please note that the MM approximation of the TMDD has been evaluated considering the dose correction for the initial condition as reported elsewhere (Yan X, Perez-Ruixo JJ, Krzyzanski W. Dose Correction for a Michaelis-Menten Approximation of a Target-Mediated Drug Disposition Model with a Multiple Intravenous Dosing Regimens. AAPS J. 2020 Jan 16;22(2):30.)

We have added the relevant references and a sentence for MM approximation as follows:

*Introduction*:

*The MM model often needs dose correction for the initial condition to prevent data misfitting and biased parameter estimates [23, 24].*

---------------------------------------------------------------------------------------------------------------------

Review references

1.	Gibiansky, L. and E. Gibiansky, *Target-mediated drug disposition model: approximations, identifiability of model parameters and applications to the population pharmacokinetic-pharmacodynamic modeling of biologics.* Expert Opin Drug Metab Toxicol, 2009. **5**(7): p. 803-12.

2.	Ma, P., *Theoretical considerations of target-mediated drug disposition models: simplifications and approximations.* Pharm Res, 2012. **29**(3): p. 866-82.

3.	Yan, X., D.E. Mager, and W. Krzyzanski, *Selection between Michaelis–Menten and target-mediated drug disposition pharmacokinetic models.* Journal of Pharmacokinetics and Pharmacodynamics, 2009. **37**(1): p. 25-47.

4.	Chetty, M., et al., *Prediction of the Pharmacokinetics, Pharmacodynamics, and Efficacy of a Monoclonal Antibody, Using a Physiologically Based Pharmacokinetic FcRn Model.* Front Immunol, 2014. **5**: p. 670.

5.	Gibiansky, L., et al., *Approximations of the target-mediated drug disposition model and identifiability of model parameters.* Journal of Pharmacokinetics and Pharmacodynamics, 2008. **35**(5): p. 573-591.

6.	Ngo, L., et al., *Development of a Pharmacokinetic Model Describing Neonatal Fc Receptor-Mediated Recycling of HL2351, a Novel Hybrid Fc-Fused Interleukin-1 Receptor Antagonist, to Optimize Dosage Regimen.* CPT Pharmacometrics Syst Pharmacol, 2020. **9**(10): p. 584-595.

7.	Lee, S.W., et al., *hIL-7-hyFc, A Long-Acting IL-7, Increased Absolute Lymphocyte Count in Healthy Subjects.* Clin Transl Sci, 2020. **13**(6): p. 1161-1169.

8.	Mager, D.E., *Target-mediated drug disposition and dynamics.* Biochemical pharmacology, 2006. **72**(1): p. 1-10.

9.	Mager, D.E. and W. Krzyzanski, *Quasi-equilibrium pharmacokinetic model for drugs exhibiting target-mediated drug disposition.* Pharmaceutical research, 2005. **22**: p. 1589-1596.

10.	Luu, K.T., et al., *A model-based approach to predicting the human pharmacokinetics of a monoclonal antibody exhibiting target-mediated drug disposition.* Journal of Pharmacology and Experimental Therapeutics, 2012. **341**(3): p. 702-708.

11.	An, G. and D.A. Katz, *Importance of Target‐Mediated Drug Disposition (TMDD) of Small‐Molecule Compounds and Its Impact on Drug Development—Example of the Class Effect of HSD‐1 Inhibitors.* The Journal of Clinical Pharmacology, 2023. **63**(5): p. 526-538.

12.	Levy, G., *Pharmacologic target‐mediated drug disposition.* Clinical Pharmacology & Therapeutics, 1994. **56**(3): p. 248-252.

13.	Dostalek, M., et al., *Pharmacokinetics, Pharmacodynamics and Physiologically-Based Pharmacokinetic Modelling of Monoclonal Antibodies.* Clinical Pharmacokinetics, 2013. **52**(2): p. 83-124.

14.	Mager, D.E. and W.J. Jusko, *General pharmacokinetic model for drugs exhibiting target-mediated drug disposition.* Journal of pharmacokinetics and pharmacodynamics, 2001. **28**: p. 507-532.

15.	Mould, D.R. and K.R. Sweeney, *The pharmacokinetics and pharmacodynamics of monoclonal antibodies-mechanistic modeling applied to drug development.* Current Opinion in Drug Discovery and Development, 2007. **10**(1): p. 84.

16.	Dua, P., E. Hawkins, and P. Van Der Graaf, *A tutorial on target‐mediated drug disposition (TMDD) models.* CPT: pharmacometrics & systems pharmacology, 2015. **4**(6): p. 324-337.

17.	Ponte, J.F., et al., *Antibody co-administration can improve systemic and local distribution of antibody–drug conjugates to increase in vivo efficacy.* Molecular cancer therapeutics, 2021. **20**(1): p. 203-212.

18.	Schauer, M. and R. Heinrich, *Quasi-steady-state approximation in the mathematical modeling of biochemical reaction networks.* Mathematical biosciences, 1983. **65**(2): p. 155-170.

19.	Sutjandra, L., et al., *Population pharmacokinetic meta-analysis of denosumab in healthy subjects and postmenopausal women with osteopenia or osteoporosis.* Clinical pharmacokinetics, 2011. **50**: p. 793-807.

20.	Radulescu, O., et al., *Reduction of dynamical biochemical reactions networks in computational biology.* Frontiers in genetics, 2012. **3**: p. 131.

21.	Grimm, H.P., *Gaining insights into the consequences of target-mediated drug disposition of monoclonal antibodies using quasi-steady-state approximations.* Journal of pharmacokinetics and pharmacodynamics, 2009. **36**: p. 407-420.

22.	Ma, P., *Theoretical Considerations of Target-Mediated Drug Disposition Models: Simplifications and Approximations.* Pharmaceutical Research, 2011. **29**(3): p. 866-882.

23.	Yan, X. and W. Krzyzanski, *Dose correction for the Michaelis–Menten approximation of the target-mediated drug disposition model.* Journal of Pharmacokinetics and Pharmacodynamics, 2012. **39**(2): p. 141-146.

24.	Yan, X., J.J.P. Ruixo, and W. Krzyzanski, *Dose Correction for a Michaelis-Menten Approximation of a Target-Mediated Drug Disposition Model with a Multiple Intravenous Dosing Regimens.* Aaps j, 2020. **22**(2): p. 30.
