# Supplementary material for: Validity conditions of approximations for a target-mediated drug disposition model: A novel first-order approximation and its comparison to other approximations
Source: PLoS Comput Biol. 2024 Apr 24;20(4):e1012066. doi: 10.1371/journal.pcbi.1012066 (PMC11090311; doi:10.1371/journal.pcbi.1012066)
Supplement: Attachment — Submitted filename: review response_r2_0327_submit.docx [file pcbi.1012066.s008.docx]

Review response for “**Validity Conditions of Approximations for a Target-Mediated Drug Disposition Model: A Novel First-Order Approximation and Its Comparison to Other Approximations”**

We thank the editor and the reviewers for their valuable feedback on our manuscript, constructive comments, and suggestions. In response to the reviewers’ comments and guidance, we have significantly edited our manuscript. Below, we give our detailed responses to the comments and describe the changes in the manuscript. Our responses are denoted in blue, while the revised portions of the manuscript are highlighted in green.

Major comments

**Reviewer #1:**

1) Terminology: I think mTMDD is the Michaelis-Menten approximation of the TMDD model. This name has a long history and is universally accepted in the pharmacometric community. The authors are free to introduce their own names and notations, but they need to provide the link to common notation and mention that mTMDD is known as Michaelis-Menten approximation as soon as they defined mTMDD. In the same spirit, total QSSA is known as QSS approximation. It is worthwhile to mention that in pharmacometrics, total QSSA is know simply as QSS.

In the abstract section, we have now revised the names of Michaelis-Menten model reduced with the standard QSSA (mTMDD) and the QSS model reduced with the total QSSA (qTMDD) as follows:

Target-mediated drug disposition (TMDD) is a phenomenon characterized by a drug's high-affinity binding to a target molecule, which significantly influences its pharmacokinetic profile within an organism. The comprehensive TMDD model delineates this interaction, yet it may become overly complex and computationally demanding in the absence of specific concentration data for the target or its complexes. Consequently, simplified TMDD models employing quasi-steady state approximations (QSSAs) have been introduced; however, the precise conditions under which these models yield accurate results require further elucidation. Here, we establish the validity of three simplified TMDD models: *the Michaelis-Menten model reduced with the* standard QSSA (mTMDD), *the QSS model reduced with* the total QSSA (qTMDD), and a first-order approximation of the total QSSA (pTMDD). Specifically, we find that mTMDD is applicable only when initial drug concentrations substantially exceed total target concentrations, while qTMDD can be used for all drug concentrations. Notably, pTMDD offers a simpler and faster alternative to qTMDD, with broader applicability than mTMDD. These findings are confirmed with antibody-drug conjugate real-world data. Our findings provide a framework for selecting appropriate simplified TMDD models while ensuring accuracy, potentially enhancing drug development and facilitating safer, more personalized treatments.

Additionally, in the author summary, we have now designated the models as the QSS model and the MM model as accepted in the pharmacometrics community as follows:

Target-mediated drug disposition (TMDD) is a phenomenon characterized by the high-affinity binding of a drug to its target molecule. The TMDD model can describe the process to elucidate the binding of the drug to its target and its elimination from the body. However, when target or complex concentrations are not available, simpler models of the TMDD model need to be used to avoid over-parameterization and to improve computational efficiency and analysis. Several simplified TMDD models based on quasi-equilibrium, *Michaelis-Menten (MM)*, or quasi-steady-state *(QSS)* *approximation* have been proposed. However, their validity conditions have not been fully investigated. In this study, we derive the validity conditions for the *approximations* of the TMDD model, providing insights into the appropriate use of simplified models. We also propose a first-order approximation of the *QSS* model, which is faster than the *QSS* model and more accurate than the *MM* model. We also applied the simplified models to antibody-drug conjugate real-world data and obtained the same results. Our work provides clear guidance on the use of the simplified TMDD models, potentially leading to improved drug development and safer, more tailored treatments for patients.

In the introduction section, we have now established the connection between the MM and QSS models through mTMDD and qTMDD, respectively, as follows:

In this study, we derived the validity criteria of approximations of the TMDD model: the MM model (referred to as mTMDD), the *QSS* model (referred to as qTMDD), and the pTMDD, which is the first-order approximation of the qTMDD model and the first derived in this study.

2) Reference Levy [1-5] is misleading. Indeed, Levi introduced the term in [5] but [1-4] are the later works of different authors. Reference [7] is the first that introduced the TMDD equations, and it is strange that it appears only as [7] rather than [2], right after reference on Levi work [5] that should be moved to [1].

Thanks for pointing this out. We have now revised the references according to the suggestion from the reviewer.

*Introduction:*

Drugs that bind to their target sites can undergo a nonlinear pharmacokinetic phenomenon that is called “target-mediated drug disposition” (TMDD), first introduced by *Levy* *(1).* TMDD occurs when the binding of a drug to its target influences the distribution and elimination of the drug, and is particularly common with biologics, such as monoclonal antibodies *(2)*. TMDD can be described with a system of ordinary differential equations for a drug, target, and drug-target complex *(3)*. The TMDD model takes into account the fact that drugs can bind to their target molecules and be eliminated from the body, or they can dissociate from their target molecules and re-enter the circulation. *This* model is critical to drug development for predicting drug efficacy and safety, selecting drug candidates, and optimizing lead compounds *(4-6)*.

3) The entire introduction with the history of TMDD and its approximation is very strange. Rather than provide logical introduction to the author’s work, it reads as an attempt to cite as many papers as possible. This is not helping the reader, but rather does not allow us to follow the author’s logic. Is it really needed for understanding the results?

References for dosing correction in the MM model were added as suggested by Reviewer 3, but we believe that they are not essential for understanding this work. We have now removed the sentence “The MM model often needs dose correction for the initial condition to prevent data misfitting and biased parameter estimate” from the introduction section.

We had added a history of Target-Mediated Drug Disposition (TMDD) as suggested by Reviewers 2 and 3. However, we have now taken into consideration Reviewer 1's comment about minimizing unnecessary citations. Therefore, we have now deleted some references to ensure better clarity and conciseness.

4) I am not sure that the new style of the paper where all the equations are moved to the end of the work is helpful. It is impossible to follow the results without seeing the formulas. I liked the prior version much more. This version is unreadable. Each statement must be accompanied by the proof, and it is impossible to follow the proof if it is separated from the results. Please return the materials and method section to their proper place after the introduction.

Following the suggestion from Reviewer 2, we relocated the mathematical parts to the materials and methods section and used the table to describe the key equations to increase the readership for those who are not familiar with mathematical analysis Furthermore, this also follows the PLoS Comp guidelines: “manuscripts should be organized as title, authors, affiliations, abstract, author summary, introduction, **results**, discussion, **materials and methods**, acknowledgments, references (Vancouver style), supporting information captions.” Thus, we have decided to keep the current format. However, we have now added the following sentence at the beginning of the results section:

*In this section, the outcomes are described without the detailed derivation of the mathematical models. We recommend reading the materials and methods section in advance for those who wish to review the detailed derivation of the models.*

5) “Materials and methods” section is better written and easier to follow. One place that is not clear is the derivation of mTMDD (lines 273-274). It seems that (2) was used as equality in derivation of mMDD from (1). Please show intermediate steps and explain the assumptions in more detail.

In the materials and methods section, we have now incorporated an additional equation, $k_{on}C\cdot R_{tot}=\left( k_{on}C+k_{off}+k_{int} \right)RC$, derived from the balanced equation, to facilitate a clear derivation of mTMDD as follows:

$$\begin{aligned} \frac{dC}{dt}=-k_{el}C-k_{on}C\cdot R_{tot}+\left( k_{on}C+k_{off} \right)RC, \#\left( 1 \right) \end{aligned}$$

MM kinetics assume that the binding rate $k_{on}C\cdot R$is balanced by the sum of dissociation and internalization $\left( k_{off}+k_{int} \right)RC$ on the scale of other processes as follows:

$\frac{C\cdot R}{RC}=\frac{k_{int}+k_{off}}{k_{on}}≝k_{m}$.

Since $R=R_{tot}-RC$, we can get

$$RC=\frac{R_{tot}\cdot C}{k_{m}+C} .$$

By substituting this to Eq. (1) *with the balanced equation,* $k_{on}C\cdot R_{tot}=\left( k_{on}C+k_{off}+k_{int} \right)RC$, we obtain mTMDD:

$$\frac{dC}{dt}=-k_{el}C-k_{int}\frac{R_{tot}\cdot C}{k_{m}+C} .$$

6) Line 281: what is mean duration of RC, how it is defined? Looks like it is similar to half-life, but without log(2).

From the equation, $RC=\frac{R_{tot}C_{0}}{C_{0}+k_{m}}\left( 1-e^{-k_{on}\left( C_{0}+k_{m} \right)t} \right)$, we note that $RC/\frac{R_{tot}C_{0}}{C_{0}+k_{m}}$ represents the cumulative distribution function of an exponential distribution. Thus, the mean duration of $RC$ is the same as in the exponential distribution with $t_{c}=\frac{1}{k_{on}\left( C_{0}+k_{m} \right)}.$

*Materials and Methods*

This model is valid when the relative change of *C* should be small during the time when *RC* approaches equilibrium ($t_{c})$, which represents initial transient time. In this case, we can estimate $t_{c}$ by substituting $C\approx C_{0}$ to Eq.(2) as follows:

$$\frac{dRC}{dt}=k_{on}C_{0}\cdot R_{tot}-\left( k_{on}C_{0}+k_{off}+k_{int} \right)RC.$$

Thus,

$$RC=\frac{R_{tot}C_{0}}{C_{0}+k_{m}}\left( 1-e^{-k_{on}\left( C_{0}+k_{m} \right)t} \right).$$

This indicates that $RC/\frac{R_{tot}C_{0}}{C_{0}+k_{m}}$ *represents the cumulative distribution function of an exponential distribution. Thus, the* mean duration of $RC$ is *the same as in the exponential distribution with* $t_{c}=1/k_{on}\left( C_{0}+k_{m} \right).$

7) The section that describes application to the real data is interesting but not clear. Where does this data come from? Were the observed data available to the authors or these were data simulated from the model? The authors should show the equations of the models fit to the data. Table 4 has parameter estimates for the linear part of the equations, but other parameters also should be shown (kon, koff, Rtot, kint, KM, kdeg, etc.). Was this population model (with inter-subject variability considered)? How many subjects were there, how well variances were estimated?

All of the datasets in the section on real-world data of antibody-drug conjugates came from clinical trial #NCT02860715 for case 1 and #NCT02175056 for case 2 as the identifier numbers for clinicaltrial.gov, respectively. One of corresponding authors (Hwi-yeol Yun) participated in both studies as a pharmacometrician, so we were able to get permission from the sponsor for their usage in our study.

We have now attached the minimal dataset used in the antibody-drug conjugate real-world data application study part. The S1 Table. Dataset for case 1 (hil-1ra-hyfc).csv file can be used for (a) the original TMDD model for case 1, (b) the mTMDD model for case 1, (c) the qTMDD model for case 1, and (d) the qTMDD model for case 1. The S2 Table. Dataset for case 2 (rhil-7-hyfc).csv file can be used for (e) the original TMDD model for case 2, (f) the mTMDD model for case 2, (g) the qTMDD model for case 2, and (h) the qTMDD model for case 2.

We have now provided additional parameters related to the TMDD phenomenon (kon, koff, Rtot, kint, KM, kdeg, etc.) as well as parameters associated with interindividual variability (Table 4) as the reviewer requested. Lastly, all model codes, made by NONMEM, were attached as additional supporting information. We have now revised the section 'Model application to real-world data of antibody-drug conjugate' in the materials and methods section and Table 4 as follows:

**Section: Materials and Methods**

**Subsection: Model application to real-world data of antibody-drug conjugates**

Two antibody-drug conjugate clinical trial studies were selected from real case application studies. Case 1 was *hIL-1Ra-hyFc* (human interleukin-1 receptor antagonist components into one antibody-derived fragment crystallizable portion) for the case of large $C_{0}/R_{tot}$(=22.33) *and case 2 was rhIL-7-hyFc (recombinant human interleukin-7, hybrid Fc-fused) for the case of small* $C_{0}/R_{tot}$*(=0.01), respectively. Specifically, the observed concentrations data were obtained from clinical trials.* See *Ngo. et al.,* (7) and *Lee et al.* (8) for detailed information on the clinical trials.

We applied the three methods (mTMDD, qTMDD and pTMDD) to the binding of a drug to its target in consideration of biological processes, and other processes were described as first-order kinetics. *In addition, population models that considered inter-individual and residual variability were conducted on the three TMDD methods, respectively. As ae general step in the population model development process, inter-individual and residual variability were explored and selected based on numerical (e.g., objective function value, OFV) and visual (e.g., goodness of fit, GoF) criteria.* Parameter optimization for the models was performed using the first-order conditional estimation with interaction (FOCE-I) method using NONMEM 7.5 and PsN 5.3.1 software, and model performance was evaluated by model diagnostics criteria and diagnostic plots. *The codes are provided in the supporting information section.*

**Table 4. Summary of the parameters estimated in the antibody-drug conjugate real-world data application study.**

| **Model** | **Case 1 for *hIL-1Ra-hyFc***  **(*C_0_/R_tot_=22.33*)** | | | | **Case 2 for r*hIL-7-hyFc***  **(*C_0_/R_tot_=0.01*)** | | | |
| --- | --- | --- | --- | --- | --- | --- | --- | --- |
|  | **TMDD** | **mTMDD** | **qTMDD** | **pTMDD** | **TMDD** | **mTMDD** | **qTMDD** | **pTMDD** |
| ***OFV*** | 1556.40 | 1564.45 | 1556.59 | 1561.90 | 1552.90 | 1611.92 | 1552.90 | 1553.14 |
| ***AIC*** | 1918.403 | 1926.45 | 1918.59 | 1923.90 | 1796.9 | 1855.92 | 1796.90 | 1797.14 |
| ***CL*** | 0.210 | 0.209 | 0.207 | 0.199 | 5.26 | 16.8 | 5.23 | 5.27 |
| ***Ka*** | 1.22 | 1.17 | 1.21 | 1.23 | 1.22(im), 0.665(sc) | 0.848(im), 0.817(sc) | 1.23(im), 0.697(sc) | 1.24(im), 0.679(sc) |
| ***Q*** | 0.0285 | 0.0145 | 0.0292 | 0.0279 | 1.21 | 73.5 | 1.21 | 1.3 |
| ***Vc*** | 11.4 | 11.6 | 11.2 | 11.3 | 1.96 | 3.96 | 1.97 | 2.07 |
| ***Vd*** | 117 | - | - | - | 31.4 | 0.00810 | 28.2 | 35.9 |
| ***KSS1*** |  | 5.97 | 233 | 162 |  | 41327.42 | 29824.48 | 29824.52 |
| ***KSS2*** |  | 67.8 | 14.1 | 14.7 |  | 29007.90* | | |
| ***Kdeg*** | 0.264* | | | | 0.642* | | | |
| ***Rtot*** | 2.23* | | | | 1060* | | | |
| ***Kuptake*** | 0.00952* | | | | 0.00952* | | | |
| ***Kint*** | 0.206* | | | | 0.642* | | | |
| ***Krecycle*** | 0.0346 | 0.0104 | 0.0332 | 0.0403 | 0.000607 | 4.68 | 0.000602 | 0.000619 |
| **Interindividual variability** | | | | | | | | |
| ***Ka*** | 0.88 | 1.21 | 0.94 | 1.01 | - | - | - | - |
| ***Krecycle*** | 0.098 | 0.398 | 0.110 | 0.0977 | - | - | - | - |
| ***Kdeg*** | 0.0851 | 0.0798 | 0.0751 | 0.0638 | - | - | - | - |
| ***CL*** | 0.0497 | 0.0389 | 0.0532 | 0.0553 | - | - | - | - |
| ***Kuptake*** | 0.886 | 0.927 | 0.813 | 0.827 | - | - | - | - |
| ***Q*** | - | - | - | - | 0.292 | 13.2 | 0.292 | 0.303 |
| **Additive error** | 0.188 | 0.237 | 0.184 | 0.185 | 0.00303 | 1.14 | 0.00348 | 0.00486 |
| **Proportional error** | 0.115 | 0.0984 | 0.115 | 0.116 | 0.332 | 0.33 | 0.332 | 0.332 |
| **Elapsed time** | 5345.33 | 403.25 | 1113.53 | 555.99 | 136.09 | 44.35 | 65.26 | 39.11 |

*OFV*: objective function value, *AIC*: Akaike information criterion, *CL*: clearance, *Ka*: absorption rate constant, *Q*: apparent inter-compartment clearance of drug, *Vc*: apparent volume of distribution (central), *Vd*: apparent volume of distribution (peripheral) , *K_SS1_*: equilibrium dissociation rate constant of drug and target binding, *K_SS2_*: equilibrium dissociation rate constant of drug and FcRn receptor binding, *K_deg_*: degradation rate of drug at distribution space, *R_tot_*: total concentration of receptor, *K_uptake_* : uptake rate of antibody, *K_int_* : internalization rate constant of FcRn-drug complex, *K_recycle_* : recycling rate constant from distribution space to a central compartment. *Those parameters were fixed based on values from the literature.

8) Overall, the manuscript has a nice idea: to introduce the new approximation as a Tailor expansion of the QSS. It has merit in some cases. Another interesting idea is to derive applicability conditions of each approximation via some regular procedure (that is not explained in sufficient detail, and thus the reader unfamiliar with the argument cannot follow it). Applications are of interest to demonstrate the place of the new approximation in the family of different simplifications of the TMDD model.

However, the manuscript in the current form is unreadable, loaded with too many references in no logical order, not well organized. My advice to the authors would be to reorganize the text in some logical order: a very brief history of the field, then TMDD equations, idea how to derive approximations and how to derive conditions for those, then the derivation of the conditions, and new approximation, and then applications (with clear statement of each problem, equations of the models, equations for the approximations, Nonmem code in supplemental materials, etc.). A short discussion may follow to outline the advantages of the new approximation, summary of conditions, etc. Discussion should mention that while the new approximation is valid for a more general case of non-constat Rtot and two-compartment model, conditions are tailored to simpler case of 1-compartment model with constant Rtot.

In summary and our response as follows:

(i) too many references in no logical order, not well organized and a very brief history of the field

Please see our responses to comments 2 and 3.

(ii) TMDD equations, idea how to derive approximations and how to derive conditions for those, then the derivation of the conditions, and new approximation,

Please see our responses to comment 4.

(iii) applications (with clear statement of each problem, equations of the models, equations for the approximations, Nonmem code in supplemental materials, etc.).

Please see our responses to comment 7.

Additionally, we have now added the NONMEM code “the population pharmacokinetic model with each approximation method” in S1 Text (Supporting information).

(iv) A short discussion may follow to outline the advantages of the new approximation, summary of conditions, etc.

We have now discussed the advantages of approximations and summary of conditions in the discussion section as follows:

*Specifically, mTMDD was derived with the balanced equation,* $k_{on}C\cdot R=\left( k_{off}+k_{int} \right)RC$*, and the total target,* $R_{tot}=R+RC$*. qTMDD was derived with the conditions of mTMDD and additional condition of the total drug,* $C_{tot}=C+RC,$ *enabling the derivation of the closed form of* $C=C(C_{tot})$*. pTMDD was derived with the Taylor expansion of* $C=C(C_{tot})$*.* We established the validity criteria of these approximations, whose accuracies are supported by both simulation and real data analysis. *Precisely, mTMDD, qTMDD, and pTMDD were validated by* $L_{m}, L_{q},$ *and* $L_{m}$ *sufficiently less than one. Additionally, we provided a simpler validity condition for the approximations by comparing* $C_{0}+k_{m}$ *and* $R_{tot}$*.* We found that pharmacokinetic models utilizing mTMDD, qTMDD, and pTMDD yielded estimates consistent with the full TMDD model when drug concentrations exceed the target. Conversely, qTMDD and pTMDD remained accurate even when target concentrations surpassed drug levels, while mTMDD did not align as closely. Notably, pTMDD offered computational efficiency without sacrificing accuracy, making it a viable option in resource-constrained scenarios. Ultimately, our research provides valuable insights into model selection and recommends appropriate approximations for scenarios with limited data availability.

(v) Discussion should mention that while the new approximation is valid for a more general case of non-constat Rtot and two-compartment model, conditions are tailored to simpler case of 1-compartment model with constant Rtot.

We have now added the sentence “This assumption simplifies the TMDD model by reducing it to two compartments ($C$ and $RC$)” in the discussion section as follows:

We assumed that the total target concentrations were constant (i.e., $k_{deg}=k_{int}$) in order to prevent the validity criteria complex. *This assumption simplifies the TMDD model by reducing it to two compartments (*$C$ *and* $RC$*).* While this assumption is commonly used (9-11), developing more flexible criteria without this assumption may offer a more comprehensive assessment of the TMDD model, facilitating better-informed decisions. We also assumed that the drug amount in the peripheral compartment was zero. Without this assumption, the peripheral compartment needs to be incorporated in Eq. (1). Future work will investigate a new approach to validity criteria in generalizing the TMDD model considering a non-zero drug amount in the peripheral compartment.

Reference

1.	Levy G. Pharmacologic target‐mediated drug disposition. Clinical Pharmacology & Therapeutics. 1994;56(3):248-52.

2.	Dostalek M, Gardner I, Gurbaxani BM, Rose RH, Chetty M. Pharmacokinetics, Pharmacodynamics and Physiologically-Based Pharmacokinetic Modelling of Monoclonal Antibodies. Clinical Pharmacokinetics. 2013;52(2):83-124.

3.	Mager DE, Jusko WJ. General pharmacokinetic model for drugs exhibiting target-mediated drug disposition. Journal of pharmacokinetics and pharmacodynamics. 2001;28:507-32.

4.	Mould DR, Sweeney KR. The pharmacokinetics and pharmacodynamics of monoclonal antibodies-mechanistic modeling applied to drug development. Current Opinion in Drug Discovery and Development. 2007;10(1):84.

5.	Luu KT, Bergqvist S, Chen E, Hu-Lowe D, Kraynov E. A model-based approach to predicting the human pharmacokinetics of a monoclonal antibody exhibiting target-mediated drug disposition. Journal of Pharmacology and Experimental Therapeutics. 2012;341(3):702-8.

6.	Ponte JF, Lanieri L, Khera E, Laleau R, Ab O, Espelin C, et al. Antibody co-administration can improve systemic and local distribution of antibody–drug conjugates to increase in vivo efficacy. Molecular cancer therapeutics. 2021;20(1):203-12.

7.	Ngo L, Oh J, Kim A, Back HM, Kang WH, Chae JW, et al. Development of a Pharmacokinetic Model Describing Neonatal Fc Receptor-Mediated Recycling of HL2351, a Novel Hybrid Fc-Fused Interleukin-1 Receptor Antagonist, to Optimize Dosage Regimen. CPT Pharmacometrics Syst Pharmacol. 2020;9(10):584-95.

8.	Lee SW, Choi D, Heo M, Shin EC, Park SH, Kim SJ, et al. hIL-7-hyFc, A Long-Acting IL-7, Increased Absolute Lymphocyte Count in Healthy Subjects. Clin Transl Sci. 2020;13(6):1161-9.

9.	Ma P. Theoretical considerations of target-mediated drug disposition models: simplifications and approximations. Pharm Res. 2012;29(3):866-82.

10.	Gibiansky L, Gibiansky E. Target-mediated drug disposition model: approximations, identifiability of model parameters and applications to the population pharmacokinetic-pharmacodynamic modeling of biologics. Expert Opin Drug Metab Toxicol. 2009;5(7):803-12.

11.	Yan X, Mager DE, Krzyzanski W. Selection between Michaelis–Menten and target-mediated drug disposition pharmacokinetic models. Journal of Pharmacokinetics and Pharmacodynamics. 2009;37(1):25-47.
